# Entropy Generation in MHD Mixed Convection Non-Newtonian Second-Grade Nanoliquid Thin Film Flow through a Porous Medium with Chemical Reaction and Stratification

**DOI:** 10.3390/e21020139

**Published:** 2019-02-01

**Authors:** Noor Saeed Khan, Zahir Shah, Saeed Islam, Ilyas Khan, Tawfeeq Abdullah Alkanhal, Iskander Tlili

**Affiliations:** 1 Department of Mathematics, Abdul Wali Khan University, Mardan 23200, Khyber Pakhtunkhwa, Pakistan; 2 Faculty of Mathematics and Statistics, Ton Duc Thang University, Ho Chi Minh City 736464, Vietnam; 3 Department of Mechatronics and System Engineering, College of Engineering, Majmaah University, Al-Majmaah 11952, Saudi Arabia; 4 Department of Mechanical and Industrial Engineering, College of Engineering, Majmaah University, Al-Majmaah 11952, Saudi Arabia

**Keywords:** entropy generation, chemical reaction, thin film, magnetohydrodynamic, gravity-driven, second-grade nanofluid, mixed convection, passively controlled nanofluid model, gyrotactic microorganisms, convective boundary conditions, residual errors, homotopy analysis method

## Abstract

Chemical reaction in mixed convection magnetohydrodynamic second grade nanoliquid thin film flow through a porous medium containing nanoparticles and gyrotactic microorganisms is considered with entropy generation. The stratification phenomena, heat and mass transfer simultaneously take place within system. Microorganisms are utilized to stabilize the suspended nanoparticles through bioconvection. For the chemical reaction of species, the mass transfer increases. The governing equations of the problem are transformed to nonlinear differential equations through similarity variables, which are solved through a well known scheme called homotopy analysis method. The solution is expressed through graphs and illustrations which show the influences of all the parameters. The residual error graphs elucidate the authentication of the present work.

## 1. Introduction

Entropy is a thermodynamic quantity representing the unavailability of a system’s thermal energy for conversion into mechanical work, often interpreted as the degree of disorder or randomness in the system. Entropy generation is an important part of the mechanical system. In the context of the present situation, irreversibility (entropy generation) is reliant to heat transmission, viscous dissipation and effects of magnetic field as well as diffusion. Irreversibility is studied with a sharp rate. The present study also discusses bioconvection and chemical reaction. Bio-convection is associated with a macroscopic flow of convecting fluid under the effect of density gradients due to hydrodynamic propulsion such as swimming of motile microorganisms. Mixed convection has several applications including heat dismissal into the atmosphere like lakes, rivers and seas. It is also utilized in the storage of thermal energy like solar ponds and heat transfer thermal sources such as the condensers of power plants. The applications of biosynthesized nanoparticles in a wide spectrum of potential areas are targeted drug delivery, cancer treatment gene therapy and DNA analysis, antibacterial agents, biosensors, enhancing reaction rates, separation science and magnetic resonance imaging (MRI). Entropy generation and bioconvection is a hot subject for scientists and researchers. Some published works and their few findings are given in Table 1.

The study of applied magnetic field plays a significant role in heat and mass transfer, controlling momentum in boundary layer nanofluid flow through a shrinking/stretching surface. Moreover, magnetohydrodynamics is utilized in diverse engineering applications like cooling of nuclear reactor, electromagnetic casting and plasma confinement. In microfluidics complexities, magnetohydrodynamics is helpful to create non-pulsating and continuous movement in the structure of complex micro-channels. In biomedical engineering, magnetohydrodynamics is applicable for magnetic drug targeting in cancer diseases. On behalf of these applications, Khan et al. [17] explored that due to the strong applied magnetic field, flow becomes slow and temperature becomes high. They also found the residual errors for the authentication of the solution. Palwasha et al. [18] discussed the intensified magnetic field which causes the Hall current effect in two dimensions case. They found that magnetic field effect is a highly dominant factor to the flow. Khan et al. [19] presented the study to show the effects of applied magnetic field on graphene nanoparticles in a thin film Eyring Powell nanofluid involving velocity slip condition. Their results reveal that the thermal conductivity increases with increasing the concentration of graphene nanoparticles. Zuhra et al. [20] also provided the study on graphene nanoparticles in two dimensional unsteady flow and heat transfer in thin liquid film non-Newtonian (Casson and Williamson) nanofluids suspension past a stretching sheet in the presence of transverse magnetic field and non-uniform heat source/sink. Their study shows that the temperature increases with temperature dependent heat source sink parameter. Khan et al. [21] analyzed the magnetohydrodynamic study in cylindrical coordinates for nanoliquid thin film using spraying phenomenon in thermal system. Apart from various effects of different parameters on different profiles, they also discussed the pressure role in the system. Focusing on the MHD flow, Khan et al. [22] presented Brownian motion and thermophoresis effects on MHD mixed convective thin film second-grade nanofluid flow with Hall effect and heat transfer past a stretching sheet, manifesting that the flow becomes three dimensional due to Hall effect.

The study of motion through a porous space is also very important in engineering and industrial processes. A porous media is a material having pores, i.e., oxides and metals, where these pores are covered with a fluid. Such type of motion has two main benefits, as its area of dissipation is higher as compared to conventional fins that improve the heat convection. Further, the regular flow of a fluid along the whole individual beads blends the fluid effectively. The nanofluid flow through a porous media reveals enhanced thermal properties, i.e., convective heat transfer coefficients as compared with a base material and higher thermal conductivity. Consequently, a porous media having nanoparticles plays a vital role in the enhancement of heat transfer and this mechanism is very important from an application and theoretical point of view. Such types of flow can be seen in filtration, oil flow, heat exchangers, drying process and geothermal systems. Moreover, nanofluid flow through a porous media has many applications in thermal engineering which are favorable to improve the thermal properties of lubricants and oil, electronic cooling equipment and vehicle cooling. Applying Navier-Stokes equations to the physical problems, Khan et al. [23] examined the important role of flow and transfer in a second-grade fluid through a porous medium past a stretching sheet in which the porous medium carries a leading role. Their results show a close agreement with the published work. It is very important to explain how nanofluid flow is possible in a porous medium. Thus, without special precautions, nanoparticles will be simply absorbed by the porous matrix. Basically, the porous matrix will work as a filter for nanoparticles. This situation has been described, explained and modeled in the paper by Wu et al. [24]. The physical situation described in this paper reveals that the work on porous media filled by nanofluids is not just a mathematical challenge, but is based on a deep physical understanding of nanofluid flows. This demonstrates that we are simulating here a real physics problem of mixed convection flow and heat transfer in a porous medium filled by a nanofluid. Kuznetsove [25] has compiled the studies on the developments of bioconvection in porous media caused by either gyrotactic or oxytactic microorganisms, with the limitation of average pore size is larger than the size of a microorganism, which allows the smooth movement of the microorganisms. Tham et al. [26] numerically studied the steady mixed convection boundary layer flow on a horizontal circular cylinder with a constant surface temperature embedded in a porous medium saturated by a nanofluid containing both nanoparticles and gyrotactic microorganisms in a stream flowing vertically upwards for both cases of a heated and cooled cylinder. The objective of that paper is to understand the behavior of nanofluid suspensions containing nanoparticles, gyrotactic microorganisms and entropy generation at the fundamental level.

Motivated from the above discussion, the present study discusses the entropy generation and mixed convection in gravity-driven MHD second grade nanofluid flow containing both nanoparticles and gyrotactic microorganisms through a porous medium with chemical reaction along a convectively heated vertical surface. Employing suitable transformations, the basic governing equations of the problem are converted to dimensionless form which have been solved using a powerful analytic tool called Homotopy Analysis Method (HAM) [27]. The influences of all the pertinents parameters on velocity, temperature, nanoparticle concentration, density motile microorganisms and entropy generation profiles have been shown graphically and illustrated.

## 2. Methods

### Basic Equations

**Figure 1:**
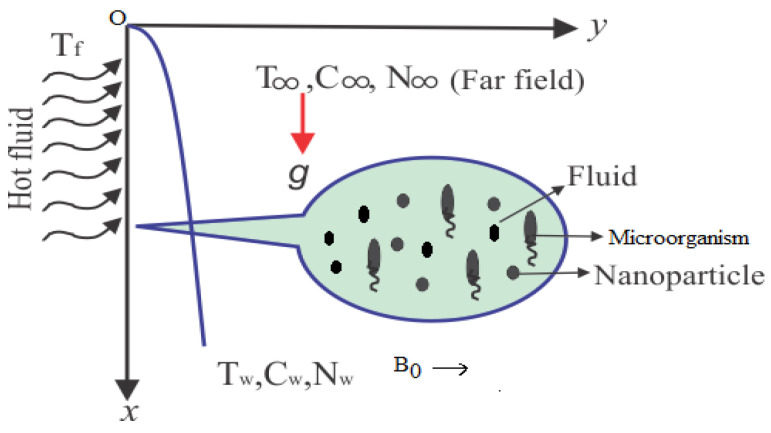
Geometry of the physical model and coordinates system.

A motion of a magnetohydrodynamic two dimensional, time independent, laminar and an incompressible second grade nanofluid falling downwards through a porous medium along a vertical solid surface due to gravity is considered. The uniform incoming flow on the right side of the plate has a constant temperature *T${}_{\infty }$* at *x* = 0 and the nanofluid on the left side of the plate has another constant temperature *T${}_{f}$*. To avoid the bioconvection instability, it is assumed that the nanofluid is dilute. In addition, the assumption is taken for the stability of the nanoparticles suspended in the base fluid so that the nanoparticles do not agglomerate in the fluid. The passive control of nanoparticles volume fraction at the boundary (on the solid wall) is taken. It is assumed that the microorganisms have constant distributions on the wall. It is most important to note that the base fluid is water so that the microorganisms can survive. The assumption is also maintained that the existence of nanoparticles has few effects on the motion of the microorganisms. A transverse magnetic field of strength *B${}_{0}$* is applied in the positive *y*-direction, normal to the plate surface. There is no applied voltage and the magnetic Reynolds number is small, so the induced magnetic field and Hall effects are negligible. The physical model and coordinate system are shown in Figure 1.

Applying the above-mentioned assumptions, the equations for the nano-bioconvection model are written in the following form as in [4,13,14,15]

(1) $$\frac{\partial u}{\partial x}+\frac{\partial v}{\partial y}\;=\;0,$$

(2) $$\begin{array}{c} \rho_{f}\left(\begin{array}{c} u\frac{\partial u}{\partial x}+v\frac{\partial u}{\partial y} \end{array}\right)\;=\;\rho_{f}U\frac{dU}{dx}+\mu_{f}\frac{\partial^{2}u}{\partial y^{2}}+\alpha_{1}\left[\begin{array}{c} \frac{\partial }{\partial x}\left(\begin{array}{c} u\frac{\partial^{2}u}{\partial y^{2}} \end{array}\right)-\frac{\partial u}{\partial y}\frac{\partial^{2}u}{\partial x\partial y}+v\frac{\partial^{3}u}{\partial y^{3}} \end{array}\right]+\left[\begin{array}{c} \left(1-C\right)\rho_{f_{\infty }}g\beta \left(T-T_{\infty }\right) \end{array}\right]\\ -\left[\begin{array}{c} \left(\rho_{p}-\rho_{f_{\infty }}\right)g\left(C-C_{\infty }\right) \end{array}\right]+\left[\begin{array}{c} g\left(N-N_{\infty }\right)\gamma_{av}\Delta \rho \end{array}\right]-\sigma B_{0}^{2}u-\frac{\nu_{f}}{k}\left(u-U_{\infty }\right)-\frac{k^{F}}{\sqrt{k}}\left(u^{2}-U_{\infty }^{2}\right), \end{array}$$

(3) $$u\frac{\partial T}{\partial x}+v\frac{\partial T}{\partial y}\;=\;\lambda \frac{\partial^{2}T}{\partial y^{2}}+\tau \left[\begin{array}{c} D_{B}\left(\begin{array}{c} \frac{\partial C}{\partial y}\frac{\partial T}{\partial y} \end{array}\right)+\left(\begin{array}{c} \frac{D_{T}}{T_{\infty }} \end{array}\right){\left(\begin{array}{c} \frac{\partial T}{\partial y} \end{array}\right)}^{2} \end{array}\right],$$

(4) $$u\frac{\partial C}{\partial x}+v\frac{\partial C}{\partial y}\;=\;\frac{D_{B}\partial^{2}C}{\partial y^{2}}+\frac{D_{T}}{T_{\infty }}\frac{\partial^{2}T}{\partial y^{2}}-k_{r}\left(C-C_{\infty }\right),$$

(5) $$u\frac{\partial N}{\partial x}+v\frac{\partial N}{\partial y}+\frac{\partial (N\overset{s}{v})}{\partial y}\;=\;D_{n}\frac{\partial^{2}N}{\partial y^{2}}.$$

Here

(6) $$\frac{dp}{dx}\;=\;\rho_{f}U\frac{dU}{dx},\;U\;=\;{\left(2ax\right)}^{\frac{1}{2}},$$

where *U* is the free stream velocity, *a* is constant, *u* and *v* are the velocity components in the *x*-and *y*-directions. $\alpha_{1}$ is the normal stress moduli, $\overset{s}{v}$ = $\left(\begin{array}{c} \frac{bW_{c}}{\Delta C} \end{array}\right)$$\frac{\partial C}{\partial y}$ is the average swimming velocity vector of the oxytactic microorganisms in which *b* is the chemotaxis constant and *W${}_{c}$* is the maximum cell swimming speed. The subscripts *p*, *f* and *f*${}_{\infty }$ denote respectively the solid particles, the nanofluid and the base fluid at far field. Δρ = $\rho_{cell}$−$\rho_{f_{\infty }}$ is the density difference between a cell and base fluid density at far field, $\mu_{f}$ is the dynamic viscosity, $\gamma_{av}$ is the average volume of microorganisms, σ is the electrical conductivity and $\rho_{f}$ is the density of the nanoliquid. $\nu_{f}$ = $\frac{\mu_{f}}{\rho_{f}}$ is the kinematic viscosity, β is the coefficient of volumetric volume expansion of a second grade nanofluid, *g* is the acceleration due to gravity, *C* is the nanopartical volume fraction, *k* = *k${}_{0}$x* is the permeability of porous medium and *k${}^{F}$* = $\frac{k_{0}^{F}}{\sqrt{x}}$ is the Forchheimer resistance factor where *k${}_{0}$* is the initial permeability. *N* is the number density of motile microorganisms, *C${}_{\infty }$* is the ambient nanofluid volume fraction, λ = $\frac{k_{1}}{\rho_{f}}$ is the thermal diffusivity of the nanofluid in which *k${}_{1}$* is the thermal conductivity, *k${}_{r}$* is the rate (constant) of chemical reaction, τ = $\frac{{\left(\rho c\right)}_{p}}{{\left(\rho c\right)}_{f}}$ is the ratio of nanoparticle heat capacity and the base fluid heat capacity, *D${}_{B}$* is the Brownian diffusion coefficient, *D${}_{n}$* is the diffusivity of microorganisms, *T${}_{\infty }$* is the ambient temperature, *D${}_{T}$* is the thermophoretic diffusion coefficient and *T* is the temperature inside the boundary layer.

The boundary conditions for the nano-bioconvection flow are

(7) $$u\;=\;0,\;v\;=\;0,\;-k_{1}\frac{\partial T}{\partial y}\;=\;h_{f}\left(T_{f}-T\right),\;D_{B}\frac{dC}{dy}+\frac{D_{T}}{T_{\infty }}\frac{dT}{dy}\;=\;0,\;N\;=\;N_{w}\;at\;y\;=\;0,$$

(8) $$u\to U\left(x\right),\;\frac{\partial u}{\partial y}\to 0,\;T\to T_{\infty },\;C\to C_{\infty },\;N\to N_{\infty }\;as\;y\to \infty ,$$

where *h${}_{f}$*(*x*) denotes the heat transfer coefficient due to *T${}_{f}$*, *N${}_{w}$* is the wall concentration of microorganisms and *N${}_{\infty }$* is the ambient concentration of microorganisms. Note that, in order to satisfy the boundary conditions at infinity, we have to set *N${}_{\infty }$* = 0.

Introducing the transformations for nondimensional variables *f*, θ, ϕ, Ω and similarity variable ζ as

(9) $$\psi \left(x,y\right)\;=\;{\left(\begin{array}{c} \frac{4U\nu_{f}x}{3} \end{array}\right)}^{\frac{1}{2}}f\left(\zeta \right),\;\theta \left(\zeta \right)\;=\;\frac{T-T_{\infty }}{T_{f}-T_{\infty }},\;\phi \;=\;\frac{C-C_{\infty }}{C_{w}-C_{\infty }},\;\Omega \;=\;\frac{N-N_{\infty }}{N_{w}-N_{\infty }},\;\zeta \;=\;{\left[\begin{array}{c} \frac{3U}{4\nu_{f}x} \end{array}\right]}^{\frac{1}{2}}y,$$

where ψ is the stream function such that *u* = $\frac{\partial \psi }{\partial y}$ and *v* = $-\frac{\partial \psi }{\partial x}$, *x* and *y* are the Cartesian coordinates along surface and normal to it. Equation (9) automatically satisfies mass conservation Equation (1). With the help of Equation (9), the Equations (2)–(5), (7) and (8) yield the following six Equations (10)–(15).

(10) $$\begin{array}{c} f^{\prime \prime \prime }+\frac{2}{3}\left(\begin{array}{c} 1-f^{\prime }2 \end{array}\right)+ff^{\prime \prime }+\gamma_{1}\left(2f^{\prime }f^{\prime \prime \prime }-f^{\prime \prime }2-3ff^{iv}\right)+Gr\theta -Nr\phi +Rb\Omega -Mf^{\prime }-\gamma_{3}f^{\prime }-\gamma_{4}f^{\prime 2}\;=\;0, \end{array}$$

(11) $$\theta^{\prime \prime }+Prf\theta^{\prime }+Nt{\left(\theta^{\prime }\right)}^{2}+Nb\theta^{\prime }\phi^{\prime }\;=\;0,$$

(12) $$\phi^{\prime \prime }+Lef\phi^{\prime }+\frac{Nt}{Nb}\theta^{\prime \prime }-\gamma_{5}\phi \;=\;0,$$

(13) $$\Omega^{\prime \prime }+Scf\Omega^{\prime }-\mathrm{Pe}\left(\phi^{\prime }\Omega^{\prime }+\phi^{\prime \prime }\Omega \right)\;=\;0,$$

(14) $$f\;=\;f^{\prime }\;=\;0,\;\theta^{\prime }\;=\;-\gamma_{2}\left(1-\theta \right),\;\mathrm{Nb}\phi^{\prime }+\mathrm{Nt}\theta^{\prime }\;=\;0,\;\Omega \;=\;1\;at\;\zeta \;=\;0,$$

(15) $$f^{\prime }\;=\;1,\;f^{\prime \prime }\;=\;0,\;\theta =\phi \;=\;\Omega \;=\;0\;at\;\zeta \;=\;\infty ,$$

where prime (${}^{\prime }$) represents the derivative with respect to ζ and the various parameters in Equations (10)–(14) are explained in Table 2. For $\gamma_{1}$ = 0, the present study corresponds to viscous nanofluid case and if $\gamma_{1}$ = $\gamma_{3}$ = $\gamma_{4}$ = $\gamma_{5}$ = *M* = 0, then the study [14] is recovered.

## 3. Entropy Generation

The subject of the present analysis has a close relation with engineering and industrial processes so it is interesting to investigate the rate of entropy generation. The volumetric entropy generation of the fluid can be written as

(16) $$\begin{array}{c} E_{gen}^{\prime \prime \prime }=\frac{\lambda }{T_{\infty }^{2}}{\left(\begin{array}{c} \frac{\partial T}{\partial y} \end{array}\right)}^{2}+\frac{\mu }{T_{\infty }}{\left(\begin{array}{c} \frac{\partial u}{\partial y} \end{array}\right)}^{2}+\frac{\mu \alpha_{1}}{T_{\infty }^{2}}\left(\begin{array}{c} u\frac{\partial u}{\partial y}\frac{\partial^{2}u}{\partial x\partial y}+v\frac{\partial u}{\partial y}\frac{\partial^{2}u}{\partial y^{2}} \end{array}\right)+\frac{\sigma B_{0}^{2}}{T_{\infty }}u^{2}+\frac{RD}{C_{\infty }}{\left(\begin{array}{c} \frac{\partial C}{\partial y} \end{array}\right)}^{2}+\\ \frac{RD}{T_{\infty }}\left[\begin{array}{c} \frac{\partial T}{\partial x}\frac{\partial C}{\partial x}+\frac{\partial T}{\partial y}\frac{\partial C}{\partial y} \end{array}\right]+\frac{RD}{N_{\infty }}{\left(\begin{array}{c} \frac{\partial N}{\partial y} \end{array}\right)}^{2}+\frac{RD}{T_{\infty }}\left[\begin{array}{c} \frac{\partial T}{\partial x}\frac{\partial N}{\partial x}+\frac{\partial T}{\partial y}\frac{\partial N}{\partial y} \end{array}\right], \end{array}$$

where *R* denotes the ideal gas constant and *D* represents the diffusivity. In Equation (16), on the right hand side, the first term represents the irreversibility due to heat transfer, the second term is entropy generation due to viscous dissipation, the third term is second grade fluid friction irreversibility, the fourth term is the local entropy generation due to the effect of magnetic field and the fifth to eighth terms are the irreversibilities due to diffusion effect. The characteristic entropy generation rate is

(17) $$E_{0}^{\prime \prime \prime }\;=\;\frac{\lambda {\left(T_{f}-T_{\infty }\right)}^{2}}{L^{2}T_{\infty }^{2}},$$

where *L* is the characteristic length. The entropy generation *E${}_{G}$* is the ratio between the entropy generation rate $E_{gen}^{\prime \prime \prime }$ and the characteristic entropy generation rate $E_{0}^{\prime \prime \prime }$ i.e.,

(18) $$E_{G}\;=\;\frac{E_{gen}^{\prime \prime \prime }}{E_{0}^{\prime \prime \prime }}$$

Applying Equation (9), the non-dimensional form of Equation (18) for entropy generation number *N${}_{G}$*(ζ) is

(19) $$\begin{array}{c} N_{G}\left(\zeta \right)\;=\;Re{\left(\theta^{\prime }\right)}^{2}+\frac{ReBr}{{\left(\theta_{w}\right)}^{2}}\left[\begin{array}{c} {\left(f^{\prime \prime }\right)}^{2} \end{array}\right]+\frac{ReBr\gamma_{1}}{{\left(\theta_{w}\right)}^{2}}\left(\begin{array}{c} f{\left(f^{\prime \prime }\right)}^{2}-3f^{\prime }f^{\prime \prime }f^{\prime \prime \prime } \end{array}\right)+\frac{ReBrM}{{\left(\theta_{w}\right)}^{2}}\left[\begin{array}{c} {\left(f^{\prime }\right)}^{2} \end{array}\right]+Re\gamma_{6}{\left(\begin{array}{c} \frac{\phi_{w}}{\theta_{w}} \end{array}\right)}^{2}{\left(\phi^{\prime }\right)}^{2}\\ +Re\gamma_{6}\left(\gamma_{7}+1\right)\left(\begin{array}{c} \frac{\phi_{w}}{\theta_{w}} \end{array}\right)\theta^{\prime }\phi^{\prime }+Re\gamma_{8}{\left(\begin{array}{c} \frac{\Omega_{w}}{\theta_{w}} \end{array}\right)}^{2}{\left(\Omega^{\prime }\right)}^{2}+Re\gamma_{8}\left(\gamma_{7}+1\right)\left(\begin{array}{c} \frac{\Omega_{w}}{\theta_{w}} \end{array}\right)\theta^{\prime }\Omega^{\prime }, \end{array}$$

where *Re* = $L^{2}\frac{3U}{4\nu_{f}}x^{\frac{-1}{4}}$ is the Reynolds number, *Br* = $\frac{\mu U^{2}}{\lambda T_{\infty }}$ is the Brinkman number, $\gamma_{6}$ = $\frac{RDC_{\infty }}{\lambda }$ is the diffusive constant parameter due to nanoparticles concentration, $\gamma_{7}$ = ${\left(\frac{y}{4x}\right)}^{2}$ is nondimensional positive number, $\gamma_{8}$ = $\frac{RDN_{\infty }}{\lambda }$ is the diffusive constant parameter due to gyrotactic microorganisms concentration, $\theta_{w}$ = $\frac{T_{f}-T_{w}}{T_{\infty }}$, $\phi_{w}$ = $\frac{C_{w}-C_{\infty }}{C_{\infty }}$ and $\Omega_{w}$ = $\frac{N_{w}-N_{\infty }}{N_{\infty }}$ are respectively the temperature, nanoparticles concentration and microorganisms concentration differences. Due to entropy generation rate, the second-grade nanofluid parameter and magnetic field parameter, here become respectively $\gamma_{1}$ = $\frac{\alpha_{1}U}{4T_{\infty }x}$ and *M* = $\frac{4\sigma \nu_{f}B_{0}^{2}x^{2}}{3\mu_{f}U^{2}}$.

## 4. Analytical Solution of the Problem by Homotopy Analysis Method

The initial approximations and the auxiliary linear operators for velocity, temperature, nanoparticles concentration and motile microorganism concentration are presented as

(20) $$\begin{array}{l} f_{0}\left(\zeta \right)\;=\;\zeta -\left[\begin{array}{c} \exp(-\zeta )-\exp(-2\zeta ) \end{array}\right],\;\theta_{0}\left(\zeta \right)\;=\;\exp\left(-\zeta \right)-\frac{\exp(-2\zeta )}{2+\gamma_{2}},\\ \phi_{0}\left(\zeta \right)\;=\;-\frac{Nt\gamma_{2}}{Nb(\gamma_{2}+2)}\exp\left(-\zeta \right),\;\Omega_{0}\left(\zeta \right)\;=\;\exp\left(-\zeta \right), \end{array}$$

(21) $$L_{f}\;=\;f^{\prime \prime \prime }-f^{\prime },\;L_{\theta }\;=\;\theta^{\prime \prime }-\theta ,\;L_{\phi }\;=\;\phi^{\prime \prime }-\phi ,\;L_{\Omega }\;=\;\Omega^{\prime \prime }-\Omega$$

The following properties are related to the linear operators

(22) $$\begin{array}{c} L_{f}\left[\begin{array}{c} C_{1}+C_{2}\exp\left(\zeta \right)+C_{3}\exp\left(-\zeta \right) \end{array}\right]\;=\;0,\;L_{\theta }\left[\begin{array}{c} C_{4}\exp\left(\zeta \right)+C_{5}\exp\left(-\zeta \right) \end{array}\right]\;=\;0,\\ L_{\phi }\left[\begin{array}{c} C_{6}\exp\left(\zeta \right)+C_{7}\exp\left(-\zeta \right) \end{array}\right]\;=\;0,\;L_{\Omega }\left[\begin{array}{c} C_{8}\exp\left(\zeta \right)+C_{9}\exp\left(-\zeta \right) \end{array}\right]\;=\;0, \end{array}$$

where *C${}_{i}$*(*i* = 1–9) are the arbitrary constants.

### 4.1. Zeroth-Order Deformation Problems

Introducing the nonlinear operator *ℵ* as

(23) $$\begin{array}{l} {ℵ}_{f}\left[f\left(\zeta ,p\right),\theta \left(\zeta ,p\right),\phi \left(\zeta ,p\right),\Omega \left(\zeta ,p\right)\right]\;=\;\frac{\partial^{3}f\left(\zeta ,p\right)}{\partial \zeta^{3}}+\frac{2}{3}\left[\begin{array}{c} 1-{\left(\begin{array}{c} \frac{\partial f(\zeta ,p)}{\partial \zeta } \end{array}\right)}^{2} \end{array}\right]+f\left(\zeta ,p\right)\frac{\partial^{2}f\left(\zeta ,p\right)}{\partial \zeta^{2}}+\\ \gamma_{1}\left[\begin{array}{c} 2\frac{\partial f(\zeta ,p)}{\partial \zeta }\frac{\partial^{3}f\left(\zeta ,p\right)}{\partial \zeta^{3}}-{\left(\begin{array}{c} \frac{\partial^{2}f\left(\zeta ,p\right)}{\partial \zeta^{2}} \end{array}\right)}^{2}-3f\left(\zeta ,p\right)\frac{\partial^{4}f\left(\zeta ,p\right)}{\partial \zeta^{4}} \end{array}\right]+Gr\theta \left(\zeta ,p\right)-Nr\phi \left(\zeta ,p\right)+Rb\Omega \left(\zeta ,p\right)-\\ M\frac{\partial f(\zeta ,p)}{\partial \zeta }-\gamma_{3}\frac{\partial f(\zeta ,p)}{\partial \zeta }-\gamma_{4}{\left(\begin{array}{c} \frac{\partial f(\zeta ,p)}{\partial \zeta } \end{array}\right)}^{2}, \end{array}$$

(24) $$\begin{array}{c} {ℵ}_{\theta }\left[f\left(\zeta ,p\right),\theta \left(\zeta ,p\right),\phi \left(\zeta ,p\right)\right]\;=\;\frac{\partial^{2}\theta \left(\zeta ,p\right)}{\partial \zeta^{2}}+Prf\left(\zeta ,p\right)\frac{\partial \theta (\zeta ,p)}{\partial \zeta }+Nt{\left(\begin{array}{c} \frac{\partial \theta (\zeta ,p)}{\partial \zeta } \end{array}\right)}^{2}+Nb\frac{\partial \theta (\zeta ,p)}{\partial \zeta }\frac{\partial \phi (\zeta ,p)}{\partial \zeta }, \end{array}$$

(25) $${ℵ}_{\phi }\left[f\left(\zeta ,p\right),\theta \left(\zeta ,p\right),\phi \left(\zeta ,p\right)\right]\;=\;\frac{\partial^{2}\phi \left(\zeta ,p\right)}{\partial \zeta^{2}}+Lef\left(\zeta ,p\right)\frac{\partial \phi (\zeta ,p)}{\partial \zeta }+\frac{Nt}{Nb}\frac{\partial^{2}\theta \left(\zeta ,p\right)}{\partial \zeta^{2}}-\gamma_{5}\phi \left(\zeta ,p\right),$$

(26) $$\begin{array}{c} {ℵ}_{\Omega }\left[f\left(\zeta ,p\right),\phi \left(\zeta ,p\right),\Omega \left(\zeta ,p\right)\right]\;=\;\frac{\partial^{2}\Omega \left(\zeta ,p\right)}{\partial \zeta^{2}}+Scf\left(\zeta ,p\right)\frac{\partial \Omega (\zeta ,p)}{\partial \zeta }-Pe\left[\begin{array}{c} \frac{\partial \phi (\zeta ,p)}{\partial \zeta }\frac{\partial \Omega (\zeta ,p)}{\partial \zeta }+\frac{\partial^{2}\phi \left(\zeta ,p\right)}{\partial \zeta^{2}}\Omega \left(\zeta ,p\right) \end{array}\right], \end{array}$$

where *p* is an embedding parameter such that *p* ∈ [0, 1].

The zeroth-order deformation problems are expressed as

(27) $$\left(1-p\right)L_{f}\left[f\left(\zeta ,p\right)-f_{0}\left(\zeta \right)\right]\;=\;ph{ℵ}_{f}\left[f\left(\zeta ,p\right),\theta \left(\zeta ,p\right),\phi \left(\zeta ,p\right),\Omega \left(\zeta ,p\right)\right],$$

(28) $$\left(1-p\right)L_{\theta }\left[\theta \left(\zeta ,p\right)-\theta_{0}\left(\zeta \right)\right]\;=\;ph{ℵ}_{\theta }\left[f\left(\zeta ,p\right),\theta \left(\zeta ,p\right),\phi \left(\zeta ,p\right)\right],$$

(29) $$\left(1-p\right)L_{\phi }\left[\phi \left(\zeta ,p\right)-\phi_{0}\left(\zeta \right)\right]\;=\;ph{ℵ}_{\phi }\left[f\left(\zeta ,p\right),\theta \left(\zeta ,p\right),\phi \left(\zeta ,p\right)\right],$$

(30) $$\left(1-p\right)L_{\Omega }\left[\Omega \left(\zeta ,p\right)-\Omega_{0}\left(\zeta \right)\right]\;=\;ph{ℵ}_{\Omega }\left[f\left(\zeta ,p\right),\phi \left(\zeta ,p\right),\Omega \left(\zeta ,p\right)\right],$$

where *h* represents the auxiliary non-zero parameter.

Equations (27)–(30) have the boundary conditions in Equations (31)–(34) respectively

(31) $$f\left(0,p\right)\;=\;0,\;f^{\prime }\left(0,p\right)\;=\;0,\;f^{\prime }\left(\infty ,p\right)\;=\;1.$$

(32) $$\theta^{\prime }\left(0,p\right)\;=\;-\gamma_{2}\left(1-\theta \left(0,p\right)\right),\;\theta \left(\infty ,p\right)\;=\;0.$$

(33) $$Nb\phi^{\prime }\left(0,p\right)+Nt\theta^{\prime }\left(0,p\right)\;=\;0,\;\phi \left(\infty ,p\right)\;=\;0.$$

(34) Ω(0,p)=1,Ω(∞,p)=0.

Assuming *p* = 0 and *p* = 1, the following results hold

(35) $$p\;=\;0\Rightarrow f\left(\zeta ,0\right)\;=\;f_{0}\left(\zeta \right)\;and\;p\;=\;1\Rightarrow f\left(\zeta ,1\right)\;=\;f\left(\zeta \right),$$

(36) $$p\;=\;0\Rightarrow \theta \left(\zeta ,0\right)\;=\;\theta_{0}\left(\zeta \right)\;and\;p\;=\;1\Rightarrow \theta \left(\zeta ,1\right)\;=\;\theta \left(\zeta \right),$$

(37) $$p\;=\;0\Rightarrow \phi \left(\zeta ,0\right)\;=\;\phi_{0}\left(\zeta \right)\;and\;p\;=\;1\Rightarrow \phi \left(\zeta ,1\right)\;=\;\phi \left(\zeta \right),$$

Similarly

(38) $$p\;=\;0\Rightarrow \Omega \left(\zeta ,0\right)\;=\;\Omega_{0}\left(\zeta \right)\;and\;p\;=\;1\Rightarrow \Omega \left(\zeta ,1\right)\;=\;\Omega \left(\zeta \right).$$

Using Taylor series expansion and Equations (27)–(30), gives the following results

(39) $$f\left(\zeta ,p\right)\;=\;f_{0}\left(\zeta \right)+\sum_{m\;=\;1}^{\infty }f_{m}\left(\zeta \right)p^{m},\;f_{m}\left(\zeta \right)\;=\;\frac{1}{m!}\frac{\partial^{m}f\left(\zeta ,p\right)}{\partial p^{m}}{|}_{p=0},$$

(40) $$\theta \left(\zeta ,p\right)\;=\;\theta_{0}\left(\zeta \right)+\sum_{m\;=\;1}^{\infty }\theta_{m}\left(\zeta \right)p^{m},\;\theta_{m}\left(\zeta \right)\;=\;\frac{1}{m!}\frac{\partial^{m}\theta \left(\zeta ,p\right)}{\partial p^{m}}{|}_{p=0},$$

(41) $$\phi \left(\zeta ,p\right)\;=\;\phi_{0}\left(\zeta \right)+\sum_{m\;=\;1}^{\infty }\phi_{m}\left(\zeta \right)p^{m},\;\phi_{m}\left(\zeta \right)\;=\;\frac{1}{m!}\frac{\partial^{m}\phi \left(\zeta ,p\right)}{\partial p^{m}}{|}_{p=0},$$

(42) $$\Omega \left(\zeta ,p\right)\;=\;\Omega_{0}\left(\zeta \right)+\sum_{m\;=\;1}^{\infty }\Omega_{m}\left(\zeta \right)p^{m},\;\Omega_{m}\left(\zeta \right)\;=\;\frac{1}{m!}\frac{\partial^{m}\Omega \left(\zeta ,p\right)}{\partial p^{m}}{|}_{p=0}.$$

The convergence of the series is closely related to *h*. The value of *h* is taken in such a way that the series in Equations (39)–(42) converge at *p* = 1, then Equations (39)–(42) result in

(43) $$f\left(\zeta \right)\;=\;f_{0}\left(\zeta \right)+\sum_{m\;=\;1}^{\infty }f_{m}\left(\zeta \right),$$

(44) $$\theta \left(\zeta \right)\;=\;\theta_{0}\left(\zeta \right)+\sum_{m\;=\;1}^{\infty }\theta_{m}\left(\zeta \right),$$

(45) $$\phi \left(\zeta \right)\;=\;\phi_{0}\left(\zeta \right)+\sum_{m\;=\;1}^{\infty }\phi_{m}\left(\zeta \right),$$

(46) $$\Omega \left(\zeta \right)\;=\;\Omega_{0}\left(\zeta \right)+\sum_{m\;=\;1}^{\infty }\Omega_{m}\left(\zeta \right).$$

### 4.2. m-th Order Deformation Problems

By taking *m* times derivative with respect to *p* of Equations ((27), (31)), ((28), (32)), ((29), (33)) and ((30), (34)), then dividing by *m*! and substituting *p* = 0 in each computation, yield the following m-th order deformation problems

(47) $$L_{f}\left[f_{m}\left(\zeta \right)-\chi_{m}f_{m-1}\left(\zeta \right)\right]\;=\;hR_{m}^{f}\left(\zeta \right),$$

(48) $$f_{m}\left(0\right)\;=\;f_{m}^{\prime }\left(0\right)\;=\;f_{m}^{\prime }\left(\infty \right)\;=\;0,$$

(49) $$\begin{array}{l} R_{m}^{f}\left(\zeta \right)\;=\;f_{m-1}^{\prime \prime \prime }+\frac{2}{3}\sum_{k\;=\;o}^{m-1}\left[\begin{array}{c} 1-f_{m-1-k}^{\prime }f_{k}^{\prime } \end{array}\right]+\sum_{k\;=\;o}^{m-1}\left[\begin{array}{c} f_{m-1-k}f_{k}^{\prime \prime } \end{array}\right]+\\ \;\gamma_{1}\sum_{k\;=\;0}^{m-1}\left[\begin{array}{c} 2f_{m-1-k}^{\prime }f_{k}^{\prime \prime \prime }-f_{m-1-k}^{\prime \prime }f_{k}^{\prime \prime }-3f_{m-1-k}f_{k}^{iv} \end{array}\right]+Gr\theta_{m-1}-Nr\phi_{m-1}+Rb\Omega_{m-1}-Mf_{m-1}^{\prime }-\\ \;\gamma_{3}f_{m-1}^{\prime }-\gamma_{4}\sum_{k\;=\;0}^{m-1}f_{m-1-k}^{\prime }f_{k}^{\prime }. \end{array}$$

(50) $$L_{\theta }\left[\theta_{m}\left(\zeta \right)-\chi_{m}\theta_{m-1}\left(\zeta \right)\right]\;=\;hR_{m}^{\theta }\left(\zeta \right),$$

(51) $$\theta_{m}^{\prime }\left(0\right)\;=\;\theta_{m}\left(\infty \right)\;=\;0,$$

(52) $$R_{m}^{\theta }\left(\zeta \right)\;=\;\theta_{m-1}^{\prime \prime }+Pr\sum_{k\;=\;o}^{m-1}f_{m-1-k}\theta_{k}^{\prime }+Nt\sum_{k\;=\;o}^{m-1}\theta_{m-1-k}^{\prime }\theta_{k}^{\prime }+Nb\sum_{k\;=\;o}^{m-1}\theta_{m-1-k}^{\prime }\phi_{k}^{\prime }.$$

(53) $$L_{\phi }\left[\phi_{m}\left(\zeta \right)-\chi_{m}\phi_{m-1}\left(\zeta \right)\right]\;=\;hR_{m}^{\phi }\left(\zeta \right),$$

(54) $$Nb\phi_{m}^{\prime }\left(0\right)+Nt\theta_{m}^{\prime }\left(0\right)\;=\;\phi_{m}\left(\infty \right)\;=\;0,$$

(55) $$R_{m}^{\phi }\left(\zeta \right)\;=\;\phi_{m-1}^{\prime \prime }+Le\sum_{k\;=\;o}^{m-1}f_{m-1-k}\phi_{k}^{\prime }+\frac{Nt}{Nb}\theta_{m-1}^{\prime \prime }-\gamma_{5}\phi_{m-1}.$$

(56) $$L_{\Omega }\left[\Omega_{m}\left(\zeta \right)-\chi_{m}\Omega_{m-1}\left(\zeta \right)\right]\;=\;hR_{m}^{\Omega }\left(\zeta \right),$$

(57) $$\Omega_{m}\left(0\right)\;=\;\Omega_{m}^{\prime }\left(\infty \right)\;=\;0,$$

(58) $$R_{m}^{\Omega }\left(\zeta \right)\;=\;\Omega_{m-1}^{\prime \prime }+Sc\sum_{k\;=\;o}^{m-1}f_{m-1-k}\Omega_{k}^{\prime }-Pe\sum_{k\;=\;o}^{m-1}\left[\begin{array}{c} \phi_{m-1-k}^{\prime }\Omega_{k}^{\prime }+\phi_{m-1-k}^{\prime \prime }\Omega_{k} \end{array}\right],$$

(59) $$\chi_{m}\;=\;\left\{\begin{array}{cc} 0, & m\;\leq \;1\\ 1, & m\;>\;1. \end{array}\right.$$

If *f${}_{m}^{*}$*(ζ), θ${}_{m}^{*}$(ζ), ϕ${}_{m}^{*}$(ζ) and Ω${}_{m}^{*}$(ζ) are the particular solutions, then the general solutions of Equations (47), (50), (54) and (56) are

(60) $$f_{m}\left(\zeta \right)\;=\;f_{m}^{*}\left(\zeta \right)+C_{1}+C_{2}\exp\left(\zeta \right)+C_{3}\exp\left(-\zeta \right),$$

(61) $$\theta_{m}\left(\zeta \right)\;=\;\theta_{m}^{*}\left(\zeta \right)+C_{4}\exp\left(\zeta \right)+C_{5}\exp\left(-\zeta \right),$$

(62) $$\phi_{m}\left(\zeta \right)\;=\;\phi_{m}^{*}\left(\zeta \right)+C_{6}\exp\left(\zeta \right)+C_{7}\exp\left(-\zeta \right),$$

(63) $$\Omega_{m}\left(\zeta \right)\;=\;\Omega_{m}^{*}\left(\zeta \right)+C_{8}\exp\left(\zeta \right)+C_{9}\exp\left(-\zeta \right).$$

## 5. Results

The non-linear differential Equations (10)–(13) with boundary conditions in Equations (14) and (15) have been solved with the help of symbolic computation software MATHEMATICA employing HAM program. The effects of embedded parameters on the velocity *f*(ζ), temperature θ(ζ), concentration ϕ(ζ), motile gyrotactic microorganisms Ω(ζ) fields and entropy generation have been plotted in Figures 6–17, 18–25, 26–35 and 36–46 respectively. Similarly the residual error graphs have been shown in Figures 54–57. The schematic illustration of the problem is seen in Figure 1. Liao [27] introduced *h* curves for the convergence of the series solutions of the problems. Therefore, the admissible *h*-curves for *f*(ζ), θ(ζ), ϕ(ζ) and Ω(ζ) are drawn in the ranges −1.3 ≤ *h* ≤ 0.0, −1.8 ≤ *h* ≤ 0.2, −2.1 ≤ *h* ≤ 0.1 and −2.0 ≤ *h* ≤ 0.0 in Figure 2, Figure 3, Figure 4 and Figure 5.

## 6. Discussion

### 6.1. Velocity Profile

**Figure 6:**
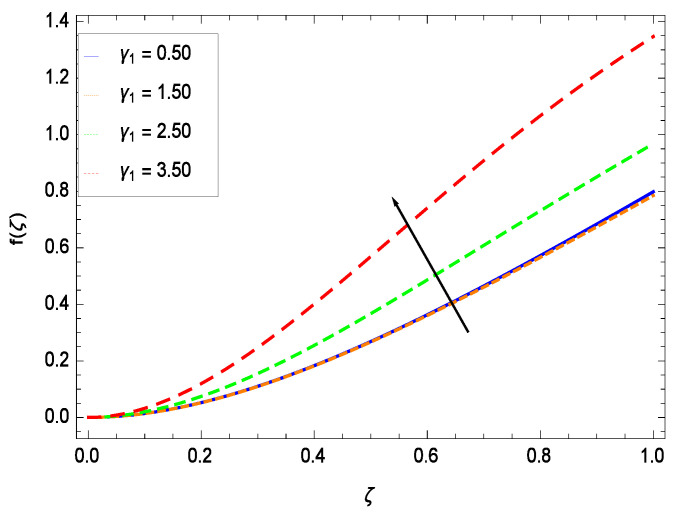
Effect on velocity profile for *h* = −1.30, $\gamma_{2}$ = 0.60, $\gamma_{3}$ = 0.20, $\gamma_{4}$ = 0.30, $\gamma_{5}$ = 1.00, *Gr* = 0.50, *Nr* = 0.60, *Rb* = 0.70, *Nb* = 0.80, *Nt* = 0.90, *Le* = 0.60, *Sc* = 0.70, *Pe* = 1.00, *M* = 1.00, *Pr* = 10.00 and different values of $\gamma_{1}$.

**Figure 7:**
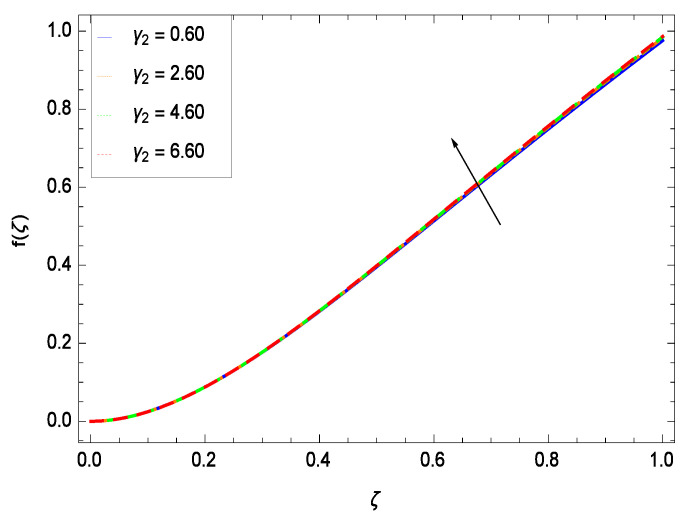
Effect on velocity profile for *h* = −1.00, $\gamma_{1}$ = 0.50, $\gamma_{3}$ = 0.20, $\gamma_{4}$ = 0.30, $\gamma_{5}$ = 1.00, *Gr* = 0.50, *Nr* = 0.60, *Rb* = 0.70, *Nb* = 0.80, *Nt* = 0.90, *Le* = 0.60, *Sc* = 0.70, *Pe* = 1.00, *M* = 1.00, *Pr* = 10.00 and different values of $\gamma_{2}$.

**Figure 8:**
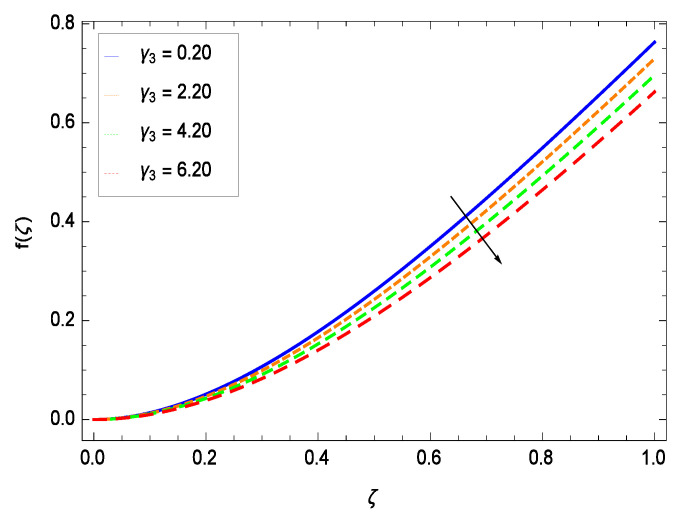
Effect on velocity profile for *h* = 0.10, $\gamma_{1}$ = 0.50, $\gamma_{2}$ = 0.60, $\gamma_{4}$ = 0.30, $\gamma_{5}$ = 1.00, *Gr* = 0.50, *Nr* = 0.60, *Rb* = 0.70, *Nb* = 0.80, *Nt* = 0.90, *Le* = 0.60, *Sc* = 0.70, *Pe* = 1.00, *M* = 1.00, *Pr* = 10.00 and different values of $\gamma_{3}$.

**Figure 9:**
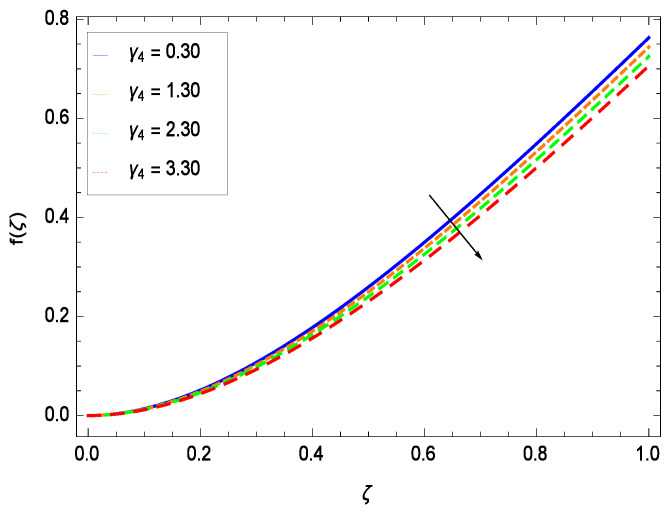
Effect on velocity profile for *h* = 0.10, $\gamma_{1}$ = 0.50, $\gamma_{3}$ = 0.20, $\gamma_{2}$ = 0.60, $\gamma_{5}$ = 1.00, *Gr* = 0.50, *Nr* = 0.60, *Rb* = 0.70, *Nb* = 0.80, *Nt* = 0.90, *Le* = 0.60, *Sc* = 0.70, *Pe* = 1.00, *M* = 1.00, *Pr* = 10.00 and different values of $\gamma_{4}$.

**Figure 10:**
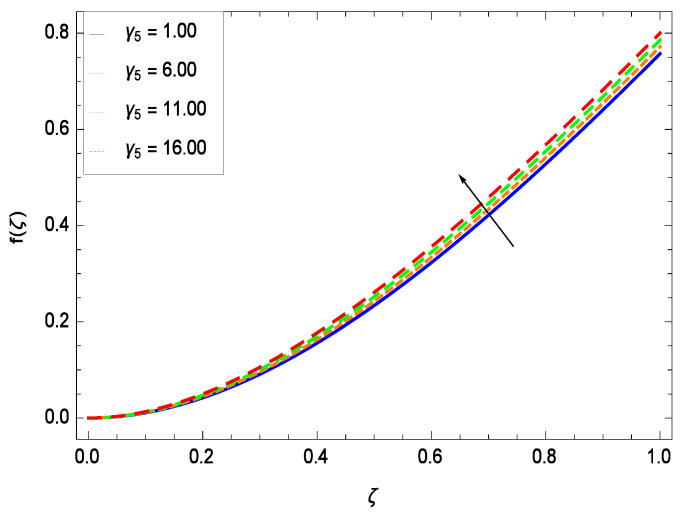
Effect on velocity profile for *h* = 0.10, $\gamma_{1}$ = 0.50, $\gamma_{3}$ = 0.20, $\gamma_{2}$ = 0.60, $\gamma_{4}$ = 0.30, *Gr* = 0.50, *Nr* = 0.60, *Rb* = 0.70, *Nb* = 0.80, *Nt* = 0.90, *Le* = 0.60, *Sc* = 0.70, *Pe* = 1.00, *M* = 1.00, *Pr* = 10.00 and different values of $\gamma_{5}$.

**Figure 11:**
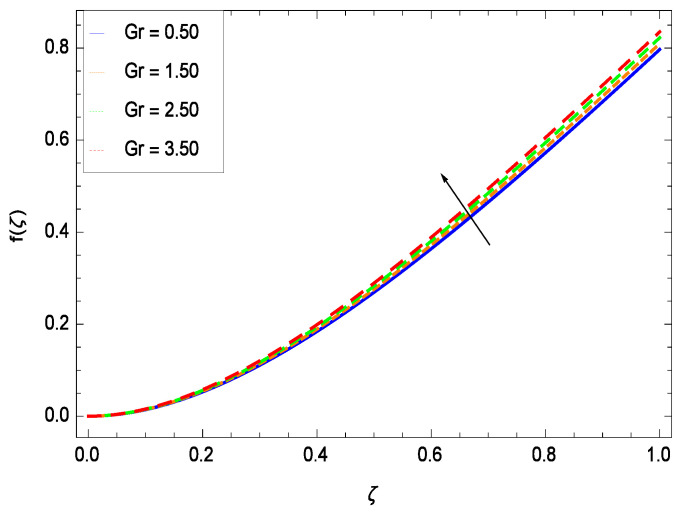
Effect on velocity profile for *h* = −1.10, $\gamma_{1}$ = 0.50, $\gamma_{2}$ = 0.60, $\gamma_{3}$ = 0.20, $\gamma_{4}$ = 0.30, $\gamma_{5}$ = 1.00, *Nr* = 0.60, *Rb* = 0.70, *Nb* = 0.80, *Nt* = 0.90, *Le* = 0.60, *Sc* = 0.70, *Pe* = 1.00, *M* = 1.00, *Pr* = 10.00 and different values of *Gr*.

**Figure 12:**
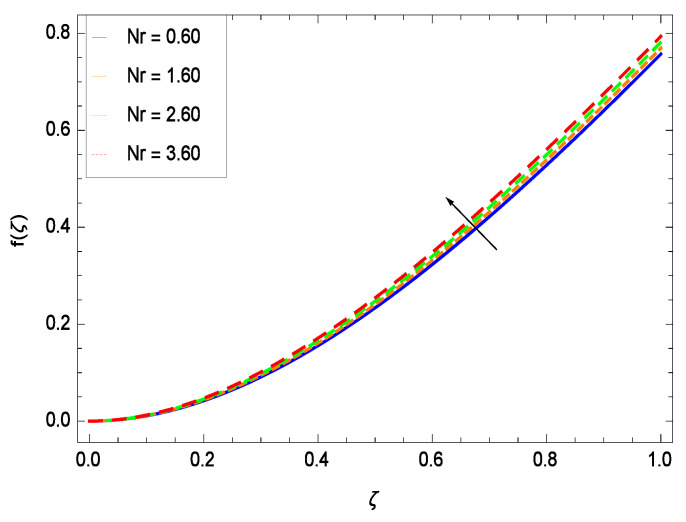
Effect on velocity profile for *h* = −3.00, $\gamma_{1}$ = 0.50, $\gamma_{2}$ = 0.60, $\gamma_{3}$ = 0.20, $\gamma_{4}$ = 0.30, $\gamma_{5}$ = 1.00, *Gr* = 0.50, *Rb* = 0.70, *Nb* = 0.80, *Nt* = 0.90, *Le* = 0.60, *Sc* = 0.70, *Pe* = 1.00, *M* = 1.00, *Pr* = 10.00 and different values of *Nr*.

**Figure 13:**
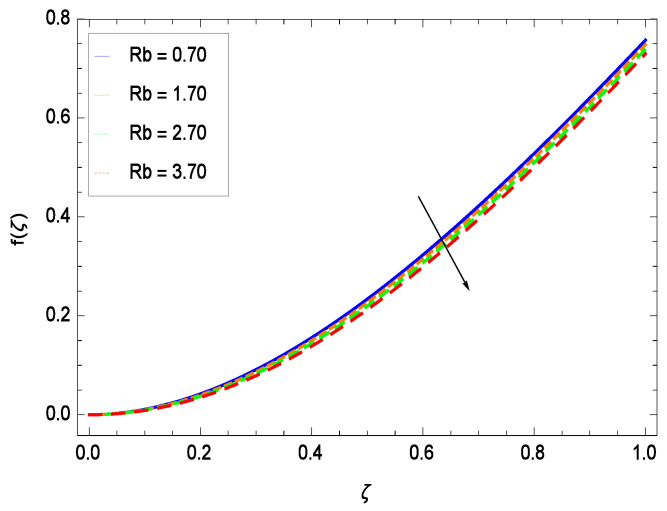
Effect on velocity profile for *h* = −3.00, $\gamma_{1}$ = 0.50, $\gamma_{2}$ = 0.60, $\gamma_{3}$ = 0.20, $\gamma_{4}$ = 0.30, $\gamma_{5}$ = 1.00, *Gr* = 0.50, *Nr* = 0.60, *Nb* = 0.80, *Nt* = 0.90, *Le* = 0.60, *Sc* = 0.70, *Pe* = 1.00, *M* = 1.00, *Pr* = 10.00 and different values of *Rb*.

**Figure 14:**
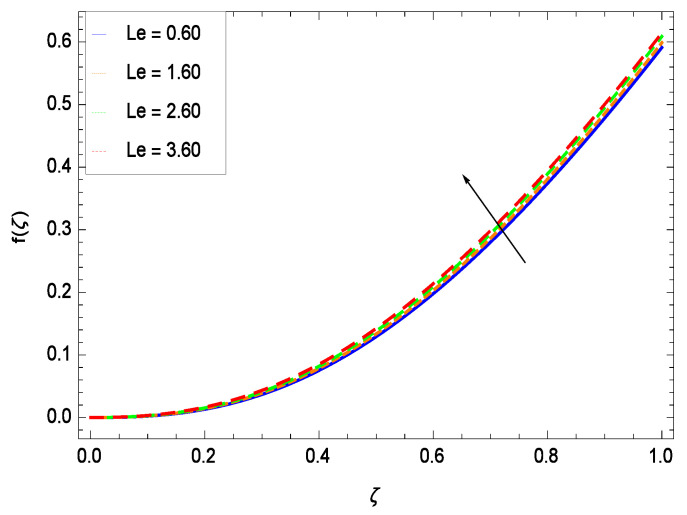
Effect on velocity profile for *h* = −5.00, $\gamma_{1}$ = 0.50, $\gamma_{2}$ = 0.60, $\gamma_{3}$ = 0.20, $\gamma_{4}$ = 0.30, $\gamma_{5}$ = 1.00, *Gr* = 0.50, *Rb* = 0.70, *Nb* = 0.80, *Nt* = 0.90, *Nr* = 0.60, *Sc* = 0.70, *Pe* = 1.00, *M* = 1.00, *Pr* = 10.00 and different values of *Le*.

**Figure 15:**
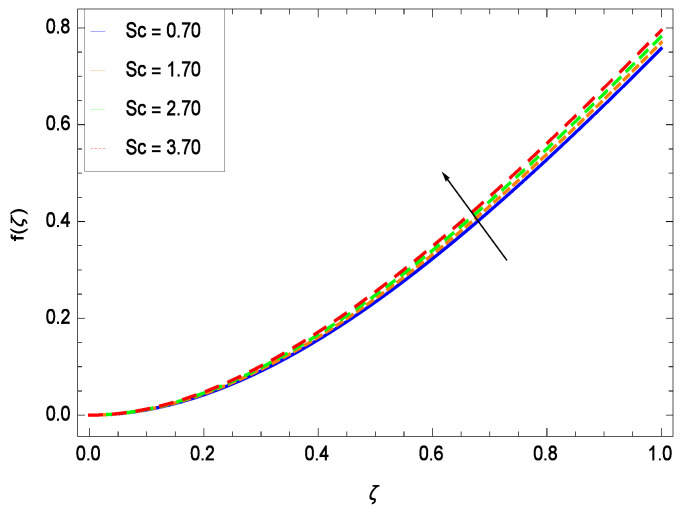
Effect on velocity profile for *h* = −3.00, $\gamma_{1}$ = 0.50, $\gamma_{2}$ = 0.60, $\gamma_{3}$ = 0.20, $\gamma_{4}$ = 0.30, $\gamma_{5}$ = 1.00, *Gr* = 0.50, *Rb* = 0.70, *Nb* = 0.80, *Nt* = 0.90, *Le* = 0.60, *Nr* = 0.60, *Pe* = 1.00, *M* = 1.00, *Pr* = 10.00 and different values of *Sc*.

**Figure 16:**
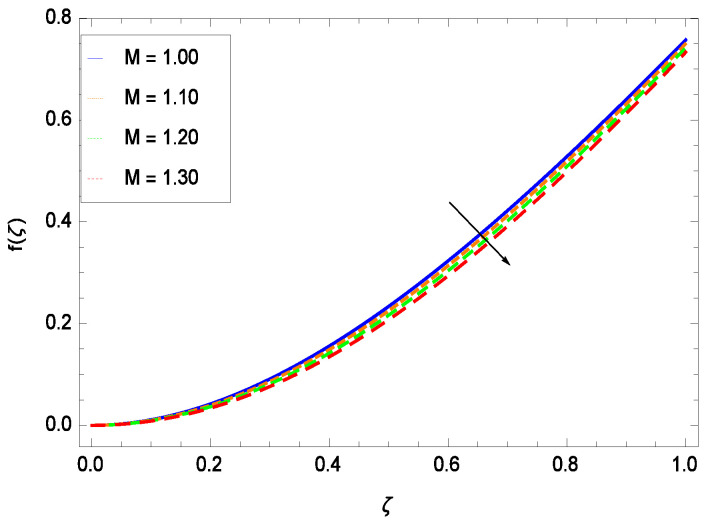
Effect on velocity profile for *h* = −3.00, $\gamma_{1}$ = 0.50, $\gamma_{2}$ = 0.60, $\gamma_{3}$ = 0.20, $\gamma_{4}$ = 0.30, $\gamma_{5}$ = 1.00, *Gr* = 0.50, *Rb* = 0.70, *Nb* = 0.80, *Nt* = 0.90, *Le* = 0.60, *Nr* = 0.60, *Pe* = 1.00, *Pr* = 10.00 and different values of *M*.

**Figure 17:**
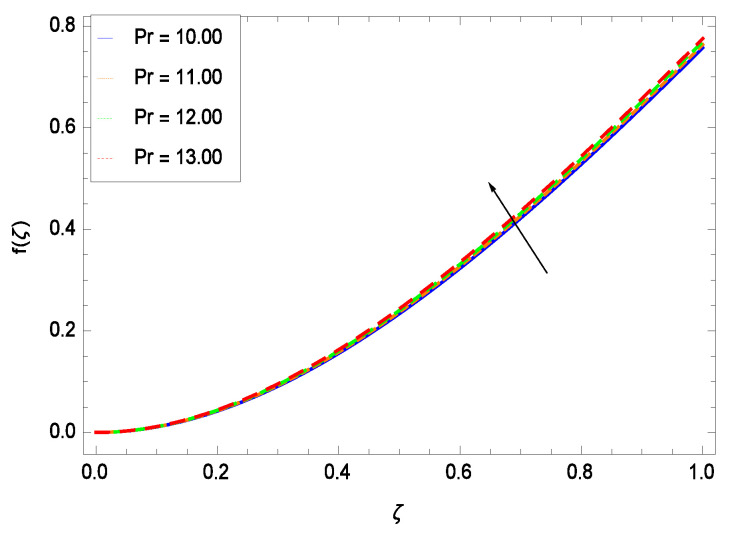
Effect on velocity profile for *h* = −3.00, $\gamma_{1}$ = 0.50, $\gamma_{2}$ = 0.60, $\gamma_{3}$ = 0.20, $\gamma_{4}$ = 0.30, $\gamma_{5}$ = 1.00, *Gr* = 0.50, *Rb* = 0.70, *Nb* = 0.80, *Nt* = 0.90, *Le* = 0.60, *Sc* = 0.70, *Pe* = 1.00, *Nr* = 0.60, *M* = 1.00 and different values of *Pr*.

This section depicts the influence of numerous dimensionless parameters on the velocity *f*(ζ). Figure 6 shows that the velocity profile decreases significantly with an enhancement in the values of second grade nanofluid parameter $\gamma_{1}$. Velocity is minimum for non-Newtonian nanofluids in general. Figure 7 demonstrates that the non-dimensional velocity profile *f*(ζ) increases for the greater values of reduced heat transfer parameter $\gamma_{2}$ due to slip condition. Figure 8 reveals that the non-dimensional velocity profile *f*(ζ) depreciates for the high values of porosity parameter $\gamma_{3}$. The holes of the medium creates resistance to the flow thereby decreasing the motion. Figure 9 is prepared for the inertial parameter $\gamma_{4}$. An increment in this parameter decreases the velocity *f*(ζ). In Figure 10, it is seen that the non-dimensional velocity *f*(ζ) amplifies with increasing magnitude of chemical reaction parameter $\gamma_{5}$ which shows that velocity enhances with the strengthening of chemical reaction. Figure 11 shows that the non-dimensional velocity *f*(ζ) increases with the increasing magnitude of buoyancy parameter *Gr*. Gravitational force favors the second grade nanofluid flow along the vertical solid surface. The buoyancy ratio parameter *Nr* is showing its effect in Figure 12. An enhancement in the parameter *Nr* makes a sharp advancement in the nanofluid flow so velocity *f*(ζ) becomes high. In Figure 13, the velocity *f*(ζ) decreases for increasing quantities of bioconvection Rayleigh number *Rb* which characterizes the effect of up-swimming of microorganisms. It should be noted that there is no effect of *Rb* on the boundary layer thickness. For the case of pure natural convection, *Rb* = 0 which implies that microorganisms have the same density as water and, therefore, they do not contribute to density stratification (or which corresponds to the situation with zero concentration of microorganisms). Since the microorganisms swim on average in the upward direction, their presence produces a destabilizing effect and reduces the critical value of *Rb*. By increasing the average concentration of corresponding microorganisms in the suspension, *Rb* can be increased. Figure 14 depicts the role of Lewis number *Le*. Velocity *f*(ζ) is found to be maximized with greater values of *Le*. Nanoparticles concentrations are consistently depressed with greater lewis number; nevertheless the over-riding influence is imposed by the lewis number on the diffusion of nanoparticles. The role of Schmidt number *Sc* is projected in Figure 15 which shows that velocity *f*(ζ) amplifies with the effective diffusion of microorganisms and the acceleration of nanoparticles. Figure 16 demonstrates that the velocity *f*(ζ) decelerates with the rising values of magnetic field parameter *M*. It is due to the fact that the magnetic field exerts a force, known as Lorentz force which suppresses the velocity. Figure 17 illustrates that the velocity *f*(ζ) enhances by the high values of the Prandtl number *Pr*. It is evident that high quantities of *Pr* make enhancement in the mass transfer rate so *f*(ζ) elevates due to gravity.

### 6.2. Temperature Profile

**Figure 18:**
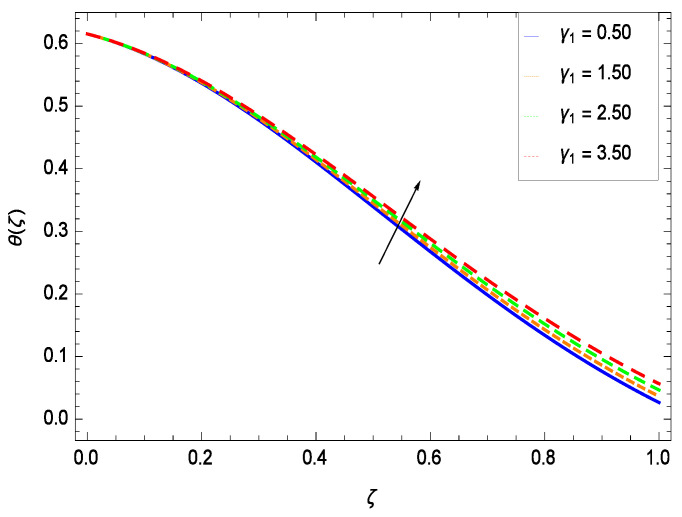
Effect on temperature profile for *h* = −0.80, $\gamma_{2}$ = 0.60, $\gamma_{3}$ = 0.20, $\gamma_{4}$ = 0.30, $\gamma_{5}$ = 1.00, *Gr* = 0.50, *Nr* = 0.60, *Rb* = 0.70, *Nb* = 0.80, *Nt* = 0.90, *Le* = 0.60, *Sc* = 0.70, *Pe* = 1.00, *M* = 1.00, *Pr* = 10.00 and different values of $\gamma_{1}$.

**Figure 19:**
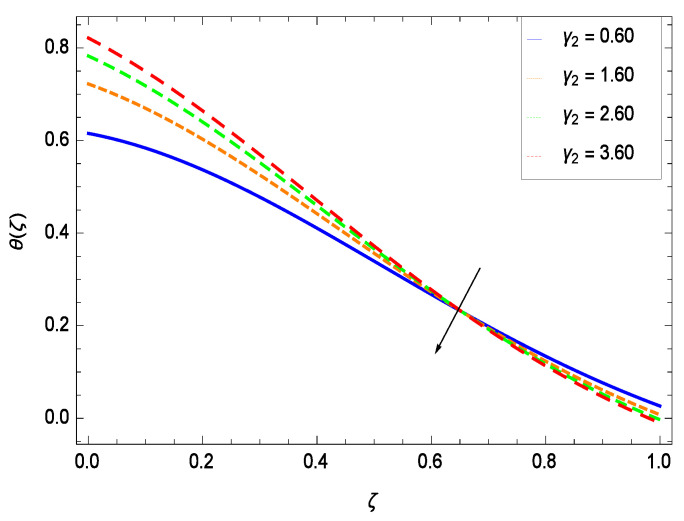
Effect on temperature profile for *h* = −0.80, $\gamma_{1}$ = 0.50, $\gamma_{3}$ = 0.20, $\gamma_{4}$ = 0.30, $\gamma_{5}$ = 1.00, *Gr* = 0.50, *Nr* = 0.60, *Rb* = 0.70, *Nb* = 0.80, *Nt* = 0.90, *Le* = 0.60, *Sc* = 0.70, *Pe* = 1.00, *M* = 1.00, *Pr* = 10.00 and different values of $\gamma_{2}$.

**Figure 20:**
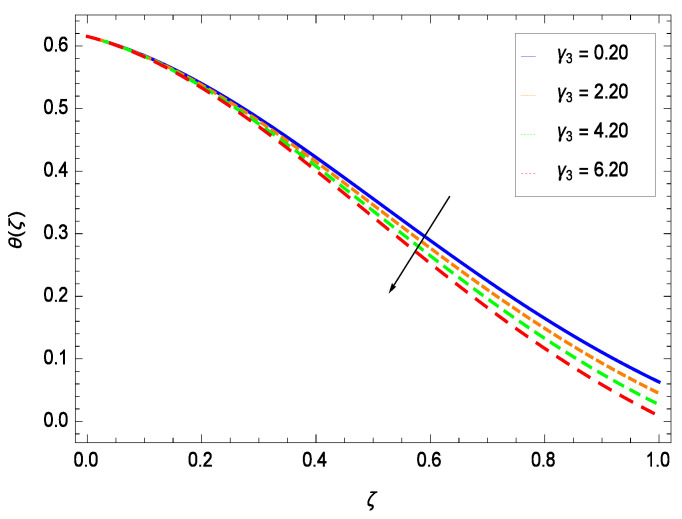
Effect on temperature profile for *h* = −0.80, $\gamma_{1}$ = 0.50, $\gamma_{2}$ = 0.60, $\gamma_{4}$ = 0.30, $\gamma_{5}$ = 1.00, *Gr* = 0.50, *Nr* = 0.60, *Rb* = 0.70, *Nb* = 0.80, *Nt* = 0.90, *Le* = 0.60, *Sc* = 0.70, *Pe* = 1.00, *M* = 1.00, *Pr* = 10.00 and different values of $\gamma_{3}$.

**Figure 21:**
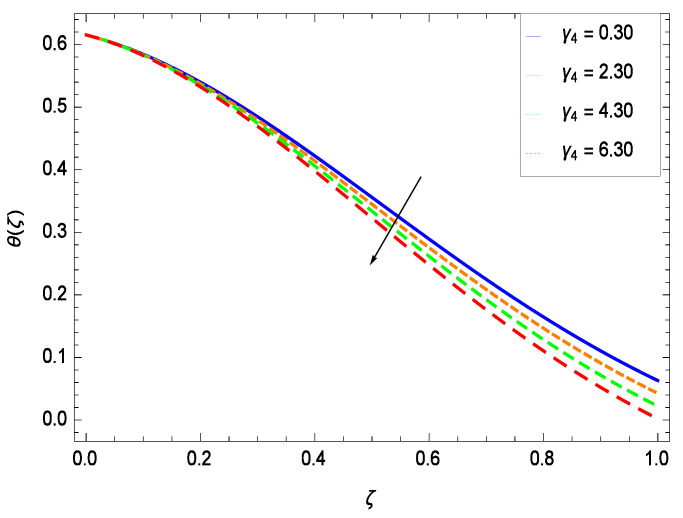
Effect on temperature profile for *h* = −0.80, $\gamma_{1}$ = 0.50, $\gamma_{3}$ = 0.20, $\gamma_{2}$ = 0.60, $\gamma_{5}$ = 1.00, *Gr* = 0.50, *Nr* = 0.60, *Rb* = 0.70, *Nb* = 0.80, *Nt* = 0.90, *Le* = 0.60, *Sc* = 0.70, *Pe* = 1.00, *M* = 1.00, *Pr* = 10.00 and different values of $\gamma_{4}$.

**Figure 22:**
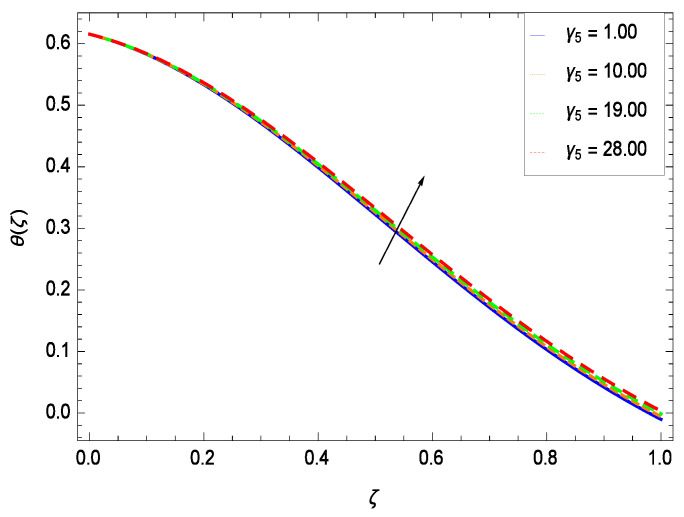
Effect on temperature profile for *h* = −0.90, $\gamma_{1}$ = 0.50, $\gamma_{3}$ = 0.20, $\gamma_{2}$ = 0.60, $\gamma_{4}$ = 0.30, *Gr* = 0.50, *Nr* = 0.60, *Rb* = 0.70, *Nb* = 0.80, *Nt* = 0.90, *Le* = 0.60, *Sc* = 0.70, *Pe* = 1.00, *M* = 1.00, *Pr* = 10.00 and different values of $\gamma_{5}$.

**Figure 23:**
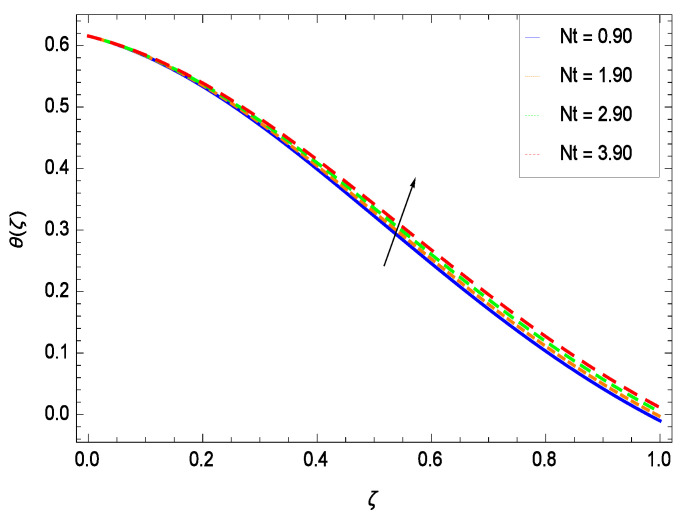
Effect on temperature profile for *h* = −0.90, $\gamma_{1}$ = 0.50, $\gamma_{2}$ = 0.60, $\gamma_{3}$ = 0.20, $\gamma_{4}$ = 0.30, $\gamma_{5}$ = 1.00, *Nr* = 0.60, *Rb* = 0.70, *Nb* = 0.80, *Gr* = 0.50, *Le* = 0.60, *Sc* = 0.70, *Pe* = 1.00, *M* = 1.00, *Pr* = 10.00 and different values of *Nt*.

**Figure 24:**
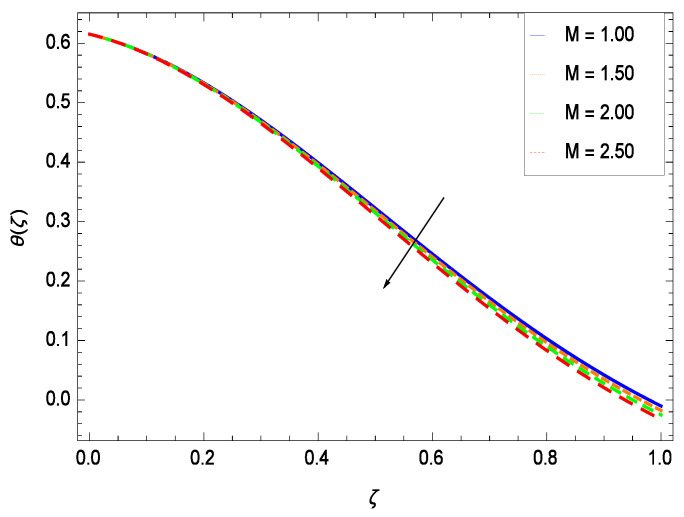
Effect on temperature profile for *h* = −0.50, $\gamma_{1}$ = 0.50, $\gamma_{2}$ = 0.60, $\gamma_{3}$ = 0.20, $\gamma_{4}$ = 0.30, $\gamma_{5}$ = 1.00, *Nr* = 0.60, *Rb* = 0.70, *Nb* = 0.80, *Nt* = 0.90, *Gr* = 0.50, *Sc* = 0.70, *Pe* = 1.00, *Pr* = 10.00 and different values of *M*.

**Figure 25:**
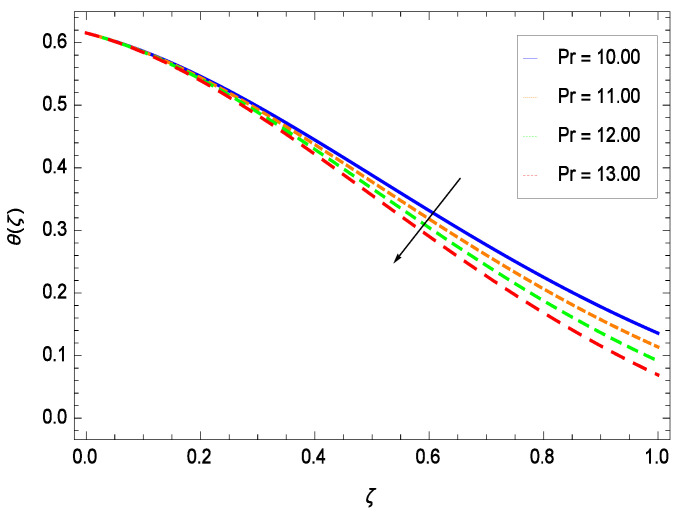
Effect on temperature profile for *h* = −0.50, $\gamma_{1}$ = 0.50, $\gamma_{2}$ = 0.60, $\gamma_{3}$ = 0.20, $\gamma_{4}$ = 0.30, $\gamma_{5}$ = 1.00, *Nr* = 0.60, *Rb* = 0.70, *Nb* = 0.80, *Nt* = 0.90, *Le* = 0.60, *Sc* = 0.70, *Pe* = 1.00, *Gr* = 0.50, *M* = 1.00 and different values of *Pr*.

Temperature profile exhibits the interesting behavior for various parameters. Figure 18 shows the influence of second grade nanofluid parameter $\gamma_{1}$. It demonstrates that the temperature θ(ζ) increases with the non-Newtonian effect of the nanofluid. The thermal slip effect is led by reduced heat transfer parameter $\gamma_{2}$ and is exhibited in Figure 19. The graph projects that with the elevation of reduced heat transfer parameter $\gamma_{2}$, temperature θ(ζ) diminishes consequently the decrement comes into play in the thermal boundary layer thickness. Figure 20 reveals that as the porosity parameter $\gamma_{3}$ increases, the surface area of the porous media increases which drops down the temperature. Figure 21 depicts that as the values of inertial parameter $\gamma_{4}$ increases, the temperature diminishes in the boundary region. Figure 22 clearly shows that temperature increases with increasing chemical reaction parameter $\gamma_{5}$. It is seen that temperature θ(ζ) increases with the sharpness of chemical reaction. Indeed, some chemical reactions like fissions and fusions make the temperature high. The thermophoresis parameter *Nt* gives rise in temperature with larger values of *Nt* as demonstrated by Figure 23. The thermophoresis parameter *Nt* is directly proportional to the heat transfer associated with the hot nanofluid. Thus an increment in the thermophoresis parameter *Nt* causes reduction in the heat transfer at the surface, consequently the temperature at the surface of the plate increases. Physically, it is due to the fact that with an enhancement of the thermophoresis parameter *Nt*, the random motion of the particles increases, resulting in an increase in the temperature profile. Due to the Brownian motion, the particles are much closer and so enhance the heat flow among the particles. Figure 24 depicts that the nondimensional temperature θ(ζ) decreases for the increasing values of magnetic field parameter *M*. It is due to the gravity because temperature rises at height and it falls down in tending towards ground. The influence of Prandtl number *Pr* has been projected in Figure 25 showing that the temperature θ(ζ) diminishes with the increasing values of *Pr*. The reason is that the increasing values of Prandtl number increase the viscosity of the fluid which absorbs the heat as a result, cooling phenomenon is generated.

### 6.3. Nanoparticle Concentration Profile

**Figure 26:**
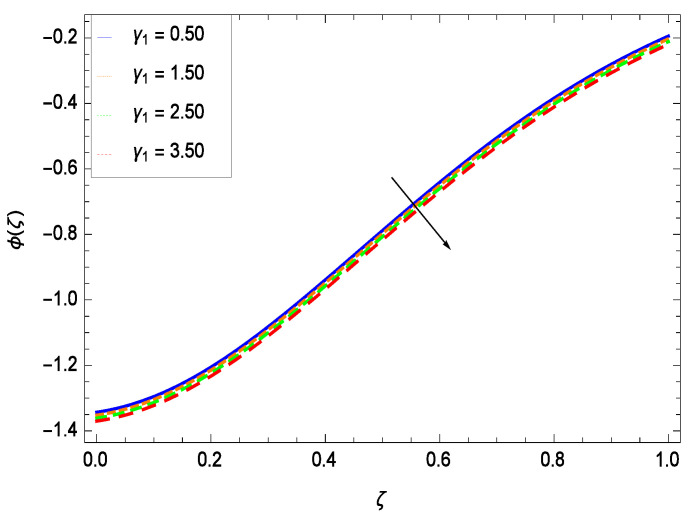
Effect on concentration profile for *h* = −3.00, $\gamma_{2}$ = 0.60, $\gamma_{3}$ = 0.20, $\gamma_{4}$ = 0.30, $\gamma_{5}$ = 1.00, *Gr* = 0.50, *Nr* = 0.60, *Rb* = 0.70, *Nb* = 0.80, *Nt* = 0.90, *Le* = 0.60, *Sc* = 0.70, *Pe* = 1.00, *M* = 1.00, *Pr* = 10.00 and different values of $\gamma_{1}$.

**Figure 27:**
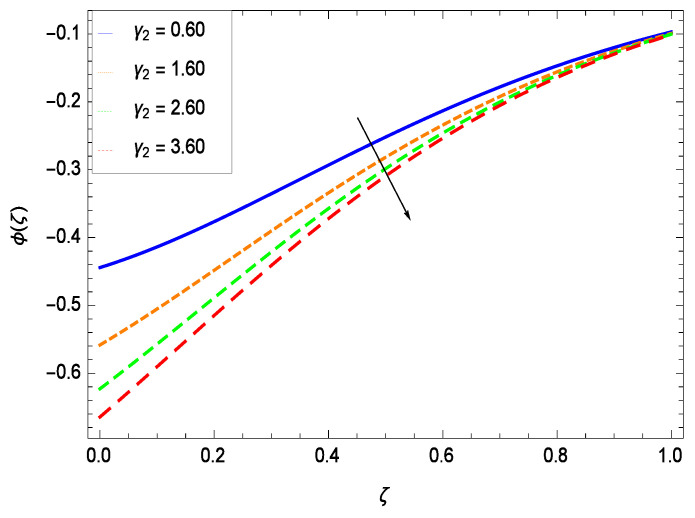
Effect on concentration profile for *h* = −3.00, $\gamma_{1}$ = 0.50, $\gamma_{3}$ = 0.20, $\gamma_{4}$ = 0.30, $\gamma_{5}$ = 1.00, *Gr* = 0.50, *Nr* = 0.60, *Rb* = 0.70, *Nb* = 0.80, *Nt* = 0.90, *Le* = 0.60, *Sc* = 0.70, *Pe* = 1.00, *M* = 1.00, *Pr* = 10.00 and different values of $\gamma_{2}$.

**Figure 28:**
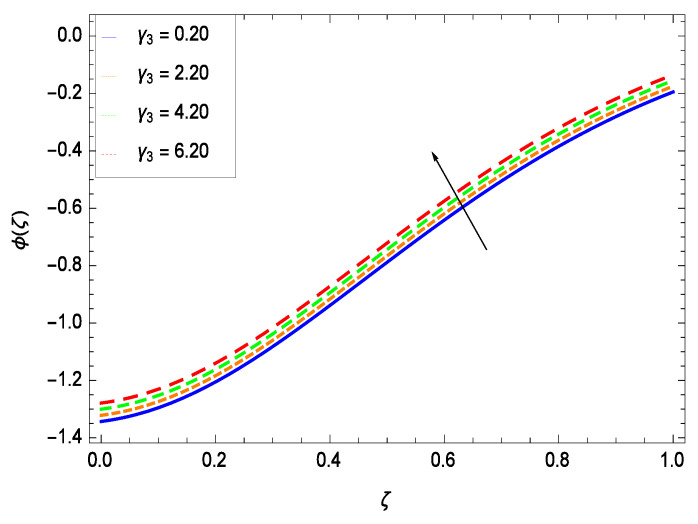
Effect on concentration profile for *h* = −3.00, $\gamma_{1}$ = 0.50, $\gamma_{2}$ = 0.60, $\gamma_{4}$ = 0.30, $\gamma_{5}$ = 1.00, *Gr* = 0.50, *Nr* = 0.60, *Rb* = 0.70, *Nb* = 0.80, *Nt* = 0.90, *Le* = 0.60, *Sc* = 0.70, *Pe* = 1.00, *M* = 1.00, *Pr* = 10.00 and different values of $\gamma_{3}$.

**Figure 29:**
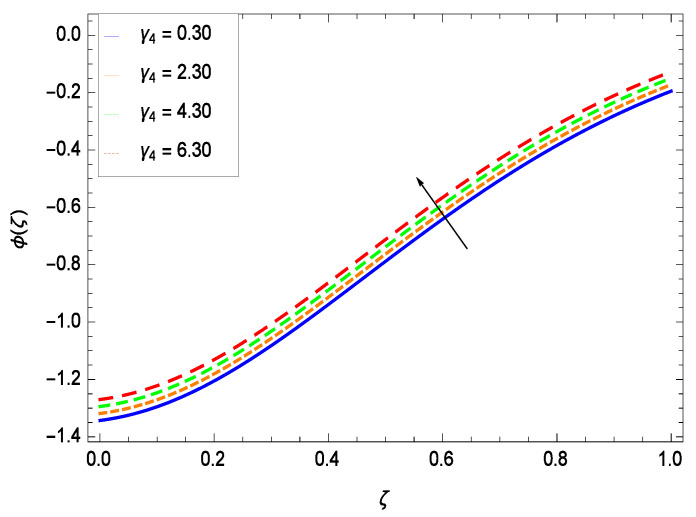
Effect on concentration profile for *h* = −3.00, $\gamma_{1}$ = 0.50, $\gamma_{3}$ = 0.20, $\gamma_{2}$ = 0.60, $\gamma_{5}$ = 1.00, *Gr* = 0.50, *Nr* = 0.60, *Rb* = 0.70, *Nb* = 0.80, *Nt* = 0.90, *Le* = 0.60, *Sc* = 0.70, *Pe* = 1.00, *M* = 1.00, *Pr* = 10.00 and different values of $\gamma_{4}$.

**Figure 30:**
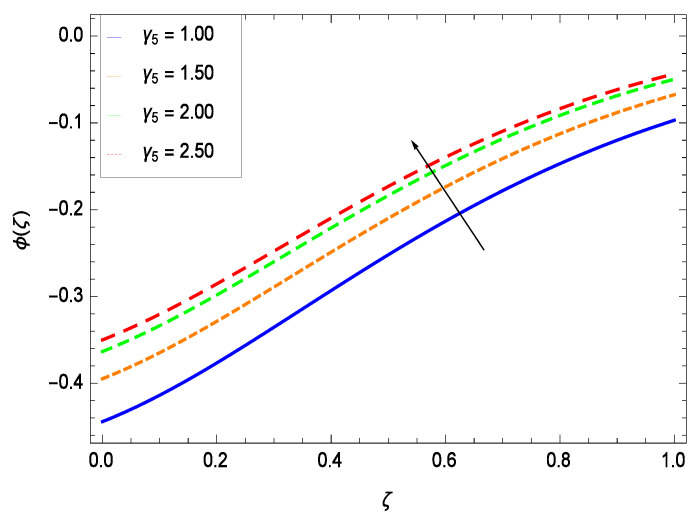
Effect on concentration profile for *h* = −1.00, $\gamma_{1}$ = 0.50, $\gamma_{3}$ = 0.20, $\gamma_{4}$ = 0.30, $\gamma_{2}$ = 0.60, *Gr* = 0.50, *Nr* = 0.60, *Rb* = 0.70, *Nb* = 0.80, *Nt* = 0.90, *Le* = 0.60, *Sc* = 0.70, *Pe* = 1.00, *M* = 1.00, *Pr* = 10.00 and different values of $\gamma_{5}$.

**Figure 31:**
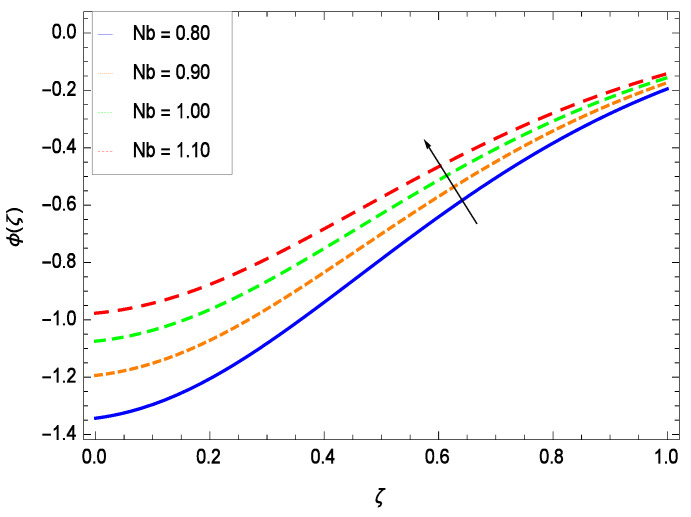
Effect on concentration profile for *h* = −3.00, $\gamma_{1}$ = 0.50, $\gamma_{2}$ = 0.60, $\gamma_{3}$ = 0.20, $\gamma_{4}$ = 0.30, $\gamma_{5}$ = 1.00, *Nr* = 0.60, *Rb* = 0.70, *Nt* = 0.90, *Le* = 0.60, *Sc* = 0.70, *Pe* = 1.00, *M* = 1.00, *Pr* = 10.00 and different values of *Nb*.

**Figure 32:**
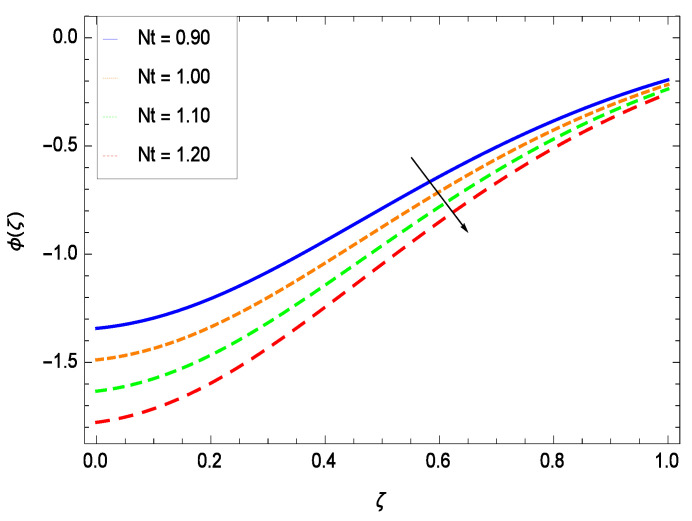
Effect on concentration profile for *h* = −.00, $\gamma_{1}$ = 0.50, $\gamma_{2}$ = 0.60, $\gamma_{3}$ = 0.20, $\gamma_{4}$ = 0.30, $\gamma_{5}$ = 1.00, *Nr* = 0.60, *Rb* = 0.70, *Nb* = 0.90, *Le* = 0.60, *Sc* = 0.70, *Pe* = 1.00, *M* = 1.00, *Pr* = 10.00 and different values of *Nt*.

**Figure 33:**
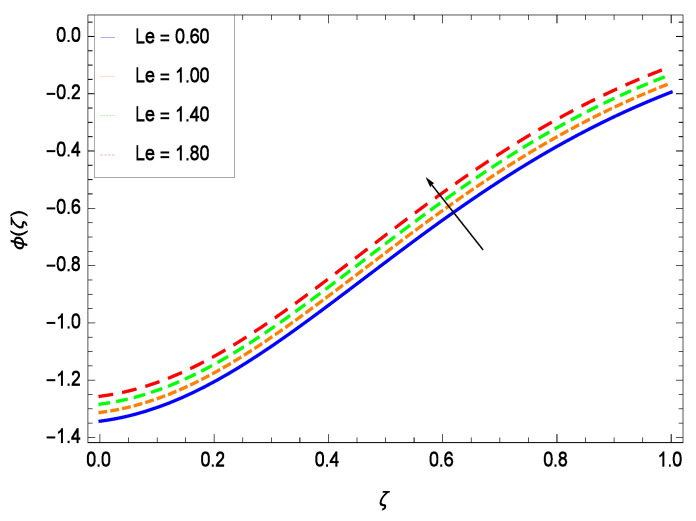
Effect on concentration profile for *h* = −3.00, $\gamma_{1}$ = 0.50, $\gamma_{2}$ = 0.60, $\gamma_{3}$ = 0.20, $\gamma_{4}$ = 0.30, $\gamma_{5}$ = 1.00, *Nr* = 0.60, *Rb* = 0.70, *Nb* = 0.80, *Nt* = 0.90, *Sc* = 0.70, *Pe* = 1.00, *M* = 1.00, *Pr* = 10.00 and different values of *Le*.

**Figure 34:**
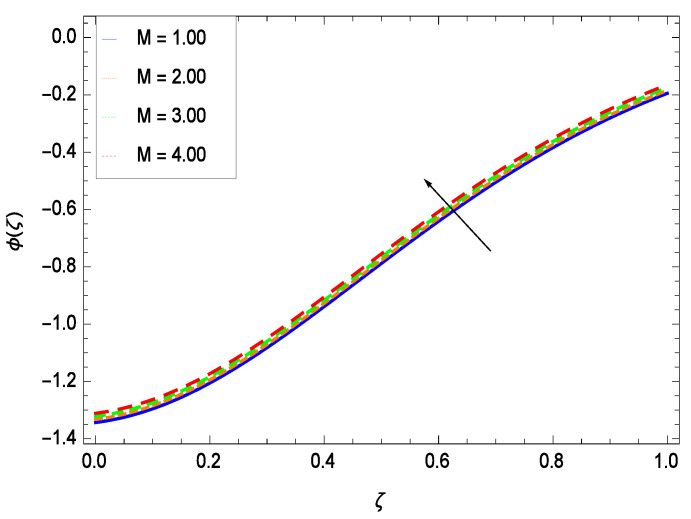
Effect on concentration profile for *h* = −3.00, $\gamma_{1}$ = 0.50, $\gamma_{2}$ = 0.60, $\gamma_{3}$ = 0.20, $\gamma_{4}$ = 0.30, $\gamma_{5}$ = 1.00, *Nr* = 0.60, *Rb* = 0.70, *Nb* = 0.80, *Nt* = 0.90, *Le* = 0.60, *Sc* = 0.70, *Pe* = 1.00, *Pr* = 10.00 and different values of *M*.

**Figure 35:**
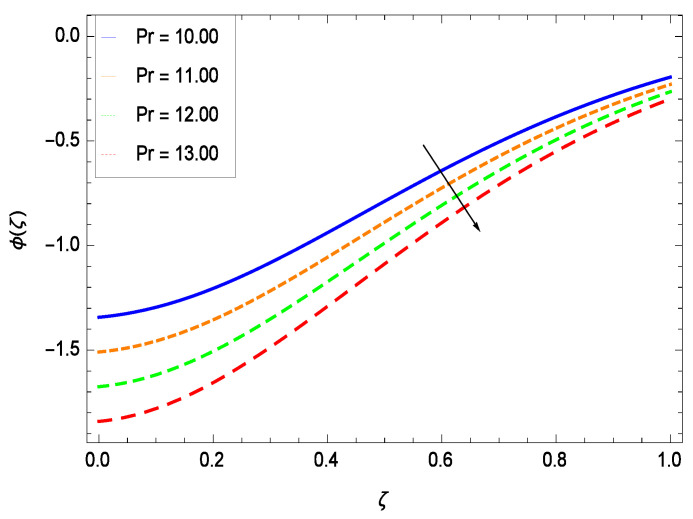
Effect on concentration profile for *h* = −3.00, $\gamma_{1}$ = 0.50, $\gamma_{2}$ = 0.60, $\gamma_{3}$ = 0.20, $\gamma_{4}$ = 0.30, $\gamma_{5}$ = 1.00, *Nr* = 0.60, *Rb* = 0.70, *Nb* = 0.80, *Nt* = 0.90, *Le* = 0.60, *Sc* = 0.70, *Pe* = 1.00, *M* = 1.00 and different values of *Pr*.

Nanoparticles having one or more dimensions of the order 100 nm or less have attracted great attention due to their unusual and fascinating properties and applications. Figure 26 displays that the concentration decreases with the non-Newtonian second grade nanofluid parameter $\gamma_{1}$. The non-Newtonian second grade nanofluid is saturated so there is less opportunity for the diffusion of the nanoparticles, therefore, the concentration decreases. Figure 27 shows the effect of reduced heat parameter $\gamma_{2}$ on concentration ϕ(ζ). It is a common phenomenon that concentration depreciates with decreasing temperature so in the present case, the concentration ϕ(ζ) falls down with decreasing the reduced heat parameter $\gamma_{2}$. Figure 28 focuses on the role of porosity parameter $\gamma_{3}$. It provides that the concentration is enhanced with the rising values of porosity parameter $\gamma_{3}$. The performance of inertial parameter $\gamma_{4}$ is shown in Figure 29 against the non-dimensional concentration profile ϕ(ζ). Concentration makes development with inertia since saturation is easily received. The highlights of the impact of chemical reaction parameter $\gamma_{5}$ is revealed in Figure 30. It is reported that for constructive (or generation) chemical reaction, the mass transfer shows an increasing behavior i.e., it is seen that the concentration profile increases significantly for the larger values of the chemical reaction parameter $\gamma_{5}$. Figure 31 projects the role of Brownian motion parameter *Nb*. Due to Brownian motion, the random motion of the particles increases, which favors the non-dimensional nanoparticle concentration profile ϕ(ζ). Figure 32 illustrates that by enhancing the thermophoresis parameter *Nt*, the concentration profile ϕ(ζ) depreciates. The nanopartical concentration boundary layer thickness is an increasing function of the thermophoresis parameter *Nt*. This indicates that an increment in the thermophoresis parameter *Nt* induces resistance to the diffusion of the solute and this assists in the reduction of the concentration gradient at the surface. It is interesting to note that the effect of the thermophoresis parameter is more pronounced for the nanoparticle volume fraction than on the fluid velocity and temperature profiles. Figure 33 is specified for the effect of Lewis number *Le* on the concentration field ϕ(ζ) which reveals that the concentration becomes rich for the different values of the Lewis number *Le*. The fact is that the performance of the nanoparticles movement is developed, which enhances the nanoparticles concentration. An increment in the Lewis number *Le* causes the reduction in the mass diffusivity of the nanofluid which in turn reduces the solute concentration. Figure 34 shows that the nondimensional concentration field ϕ(ζ) increases as the magnetic field parameter *M* increases. It is due to the existence of Lorentz force which shows resistivity to the over all motion, consequently, nanoparticle concentration increases. The magnetic field parameter increases the friction between nanofluid layers so the concentration boundary layer thickness gets enhanced. The influence of Prandtl number *Pr* on the nanoparticle concentration profile ϕ(ζ) is depicted in Figure 35. Prandtl number is the fluid viscosity parameter so by increasing Prandtl number, the internal viscous forces of the fluid is strong and the concentration goes to peak which causes no special capability for further nanoparticles concentration. Therefore, concentration ϕ(ζ) becomes weak with larger values of *Pr*.

### 6.4. Gyrotactic Microorganism Concentration

**Figure 36:**
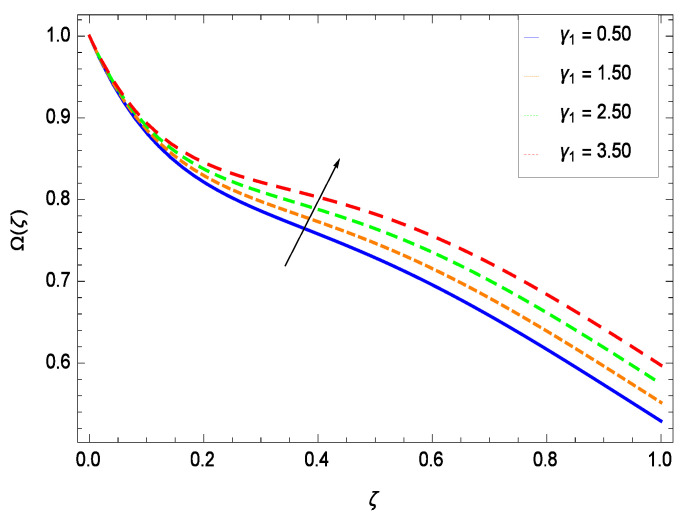
Effect on microorganisms density function for *h* = −3.00, $\gamma_{2}$ = 0.60, $\gamma_{3}$ = 0.20, $\gamma_{4}$ = 0.30, $\gamma_{5}$ = 1.00, *Gr* = 0.50, *Nr* = 0.60, *Rb* = 0.70, *Nb* = 0.80, *Nt* = 0.90, *Le* = 0.60, *Sc* = 0.70, *Pe* = 1.00, *M* = 1.00, *Pr* = 10.00 and different values of $\gamma_{1}$.

**Figure 37:**
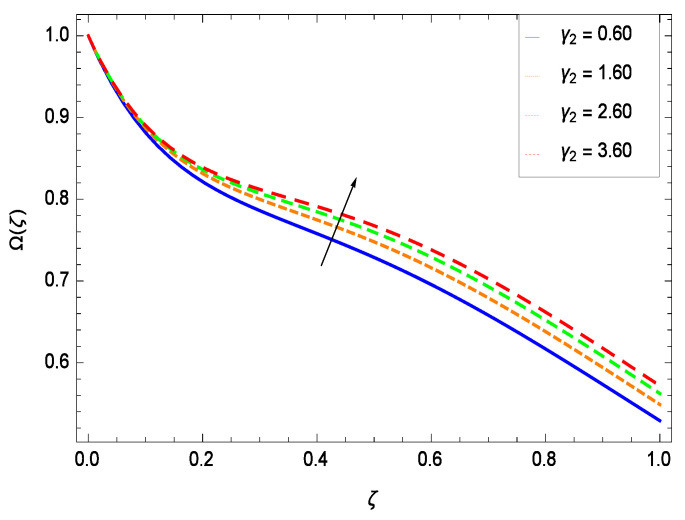
Effect on microorganisms density function for *h* = −3.00, $\gamma_{1}$ = 0.50, $\gamma_{3}$ = 0.20, $\gamma_{4}$ = 0.30, $\gamma_{5}$ = 1.00, *Gr* = 0.50, *Nr* = 0.60, *Rb* = 0.70, *Nb* = 0.80, *Nt* = 0.90, *Le* = 0.60, *Sc* = 0.70, *Pe* = 1.00, *M* = 1.00, *Pr* = 10.00 and different values of $\gamma_{2}$.

**Figure 38:**
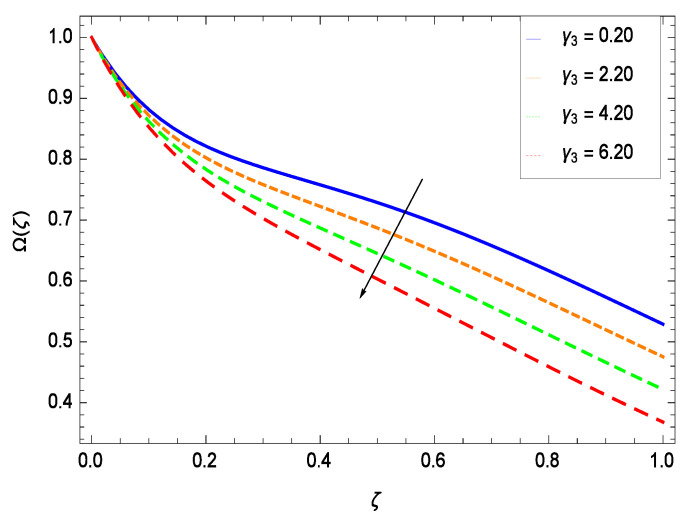
Effect on microorganisms density function for *h* = −3.00, $\gamma_{1}$ = 0.50, $\gamma_{2}$ = 0.60, $\gamma_{4}$ = 0.30, $\gamma_{5}$ = 1.00, *Gr* = 0.50, *Nr* = 0.60, *Rb* = 0.70, *Nb* = 0.80, *Nt* = 0.90, *Le* = 0.60, *Sc* = 0.70, *Pe* = 1.00, *M* = 1.00, *Pr* = 10.00 and different values of $\gamma_{3}$.

**Figure 39:**
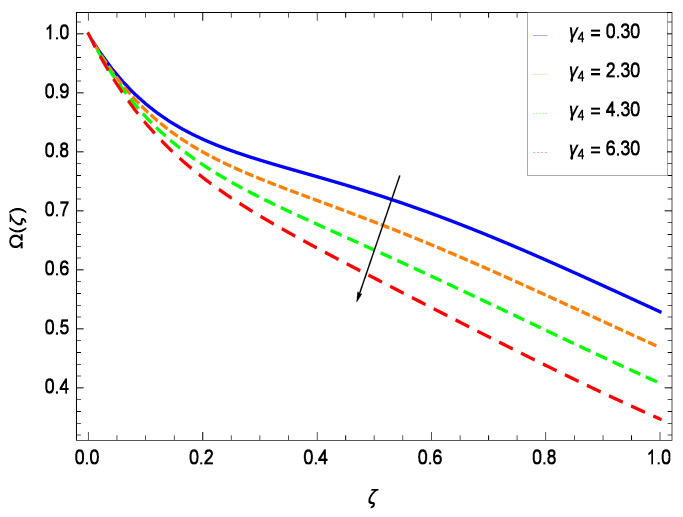
Effect on microorganisms density function for *h* = −3.00, $\gamma_{1}$ = 0.50, $\gamma_{3}$ = 0.20, $\gamma_{2}$ = 0.60, $\gamma_{5}$ = 1.00, *Gr* = 0.50, *Nr* = 0.60, *Rb* = 0.70, *Nb* = 0.80, *Nt* = 0.90, *Le* = 0.60, *Sc* = 0.70, *Pe* = 1.00, *M* = 1.00, *Pr* = 10.00 and different values of $\gamma_{4}$.

**Figure 40:**
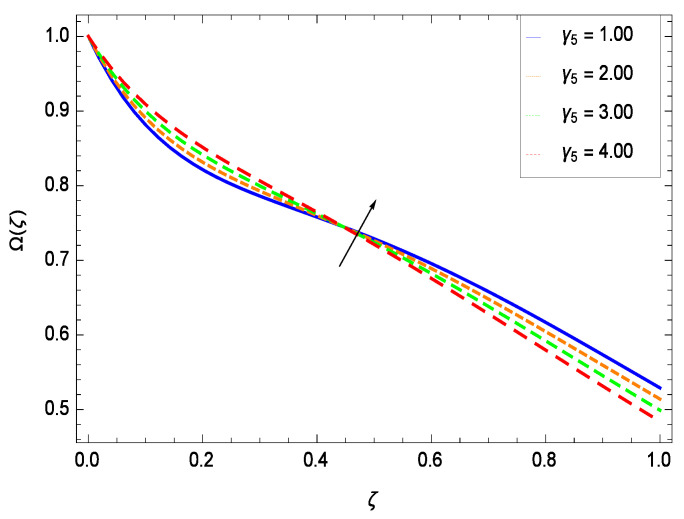
Effect on microorganisms density function for *h* = −3.00, $\gamma_{1}$ = 0.50, $\gamma_{3}$ = 0.20, $\gamma_{2}$ = 0.60, $\gamma_{4}$ = 0.30, *Gr* = 0.50, *Nr* = 0.60, *Rb* = 0.70, *Nb* = 0.80, *Nt* = 0.90, *Le* = 0.60, *Sc* = 0.70, *Pe* = 1.00, *M* = 1.00, *Pr* = 10.00 and different values of $\gamma_{5}$.

**Figure 41:**
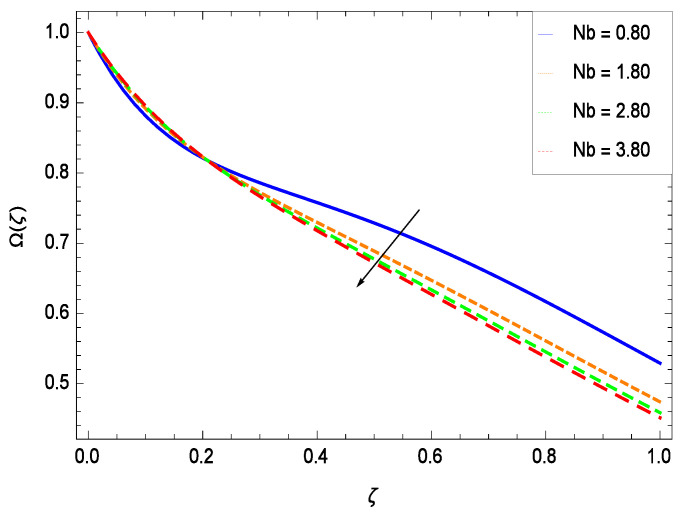
Effect on microorganisms density function for *h* = −3.00, $\gamma_{1}$ = 0.50, $\gamma_{2}$ = 0.60, $\gamma_{3}$ = 0.20, $\gamma_{4}$ = 0.30, $\gamma_{5}$ = 1.00, *Gr* = 0.50, *Nr* = 0.60, *Rb* = 0.70, *Nt* = 0.90, *Le* = 0.60, *Sc* = 0.70, *Pe* = 1.00, *M* = 1.00, *Pr* = 10.00 and different values of *Nb*.

**Figure 42:**
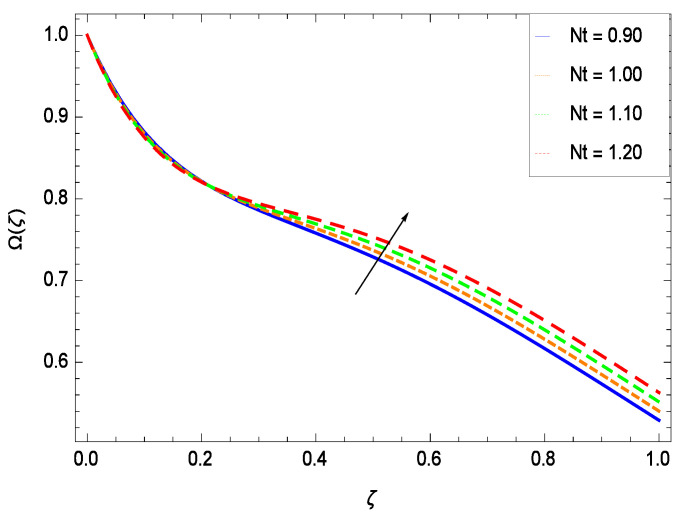
Effect on microorganisms density function for *h* = −3.00, $\gamma_{1}$ = 0.50, $\gamma_{2}$ = 0.60, $\gamma_{3}$ = 0.20, $\gamma_{4}$ = 0.30, $\gamma_{5}$ = 1.00, *Gr* = 0.50, *Nr* = 0.60, *Rb* = 0.70, *Nb* = 0.80, *Le* = 0.60, *Sc* = 0.70, *Pe* = 1.00, *M* = 1.00, *Pr* = 10.00 and different values of *Nt*.

**Figure 43:**
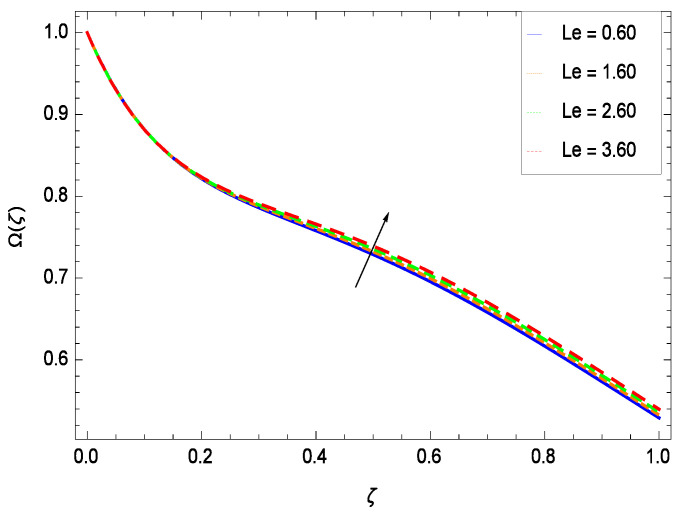
Effect on microorganisms density function for *h* = −3.00, $\gamma_{1}$ = 0.50, $\gamma_{2}$ = 0.60, $\gamma_{3}$ = 0.20, $\gamma_{4}$ = 0.30, $\gamma_{5}$ = 1.00, *Gr* = 0.50, *Nr* = 0.60, *Rb* = 0.70, *Nt* = 0.90, *Nb* = 0.80, *Sc* = 0.70, *Pe* = 1.00, *M* = 1.00, *Pr* = 10.00 and different values of *Le*.

**Figure 44:**
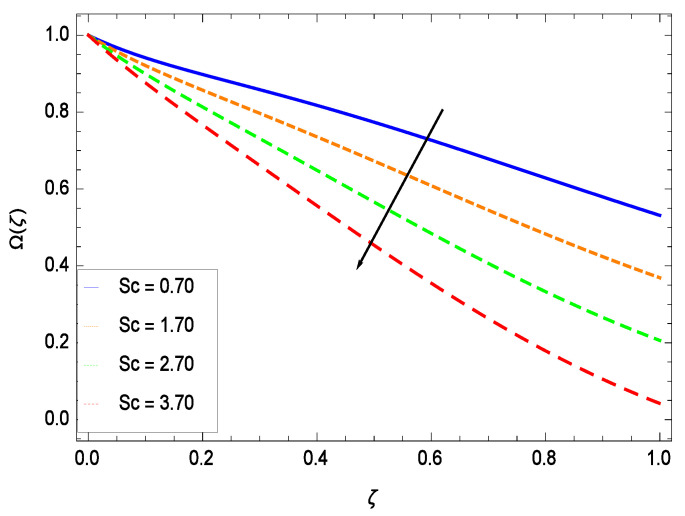
Effect on microorganisms density function for *h* = −3.00, $\gamma_{1}$ = 0.50, $\gamma_{2}$ = 0.60, $\gamma_{3}$ = 0.20, $\gamma_{4}$ = 0.30, $\gamma_{5}$ = 1.00, *Gr* = 0.50, *Nr* = 0.60, *Nb* = 0.80, *Nt* = 0.90, *Le* = 0.60, *Pe* = 1.00, *M* = 1.00, *Pr* = 10.00 and different values of *Sc*.

**Figure 45:**
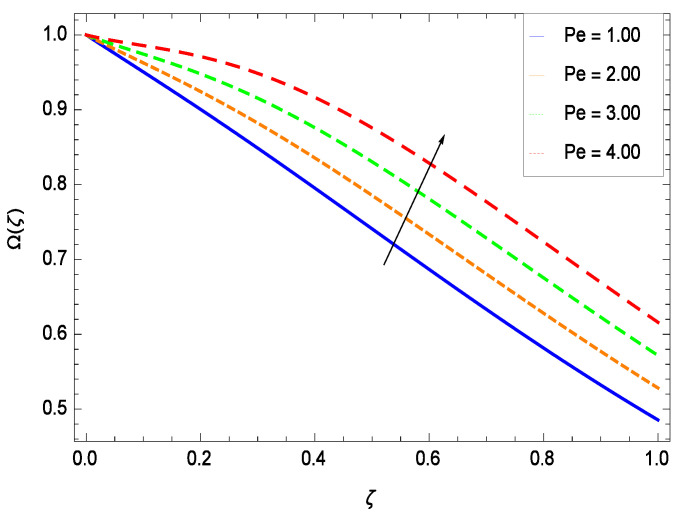
Effect on microorganisms density function for *h* = −1.10, $\gamma_{1}$ = 0.50, $\gamma_{2}$ = 0.60, $\gamma_{3}$ = 0.20, $\gamma_{4}$ = 0.30, $\gamma_{5}$ = 1.00, *Gr* = 0.50, *Nr* = 0.60, *Rb* = 0.70, *Nt* = 0.90, *Sc* = 0.70, *Nb* = 0.80, *M* = 1.00, *Pr* = 10.00 and different values of *Pe*.

**Figure 46:**
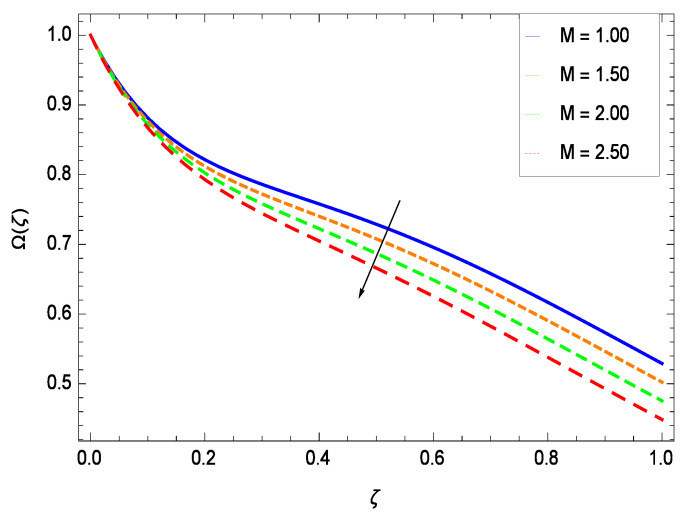
Effect on microorganisms density function for *h* = −3.00, $\gamma_{1}$ = 0.50, $\gamma_{2}$ = 0.60, $\gamma_{3}$ = 0.20, $\gamma_{4}$ = 0.30, $\gamma_{5}$ = 1.00, *Gr* = 0.50, *Nr* = 0.60, *Rb* = 0.70, *Nt* = 0.90, *Sc* = 0.70, *Nb* = 0.80, *Pe* = 1.00, *Pr* = 10.00 and different values of *M*.

There have been tremendous developments in the field of microorganism produced nanoparticles and their applications. The characteristics of the density motile microorganisms with respect to different parameters are discussed in the following. Figure 36 elucidates that the microorganism concentration profile Ω(ζ) grows up with increasing magnitude of second grade nanofluid parameter $\gamma_{1}$. The reason is that increasing the quantity $\gamma_{1}$ increases the microorganism propulsion by providing the best platform for the growth of microorganism and hence explains the reason why the microorganism concentration boundary layer of second grade nanofluid enhances. Figure 37 shows the influence of reduced heat parameter $\gamma_{2}$ on the microorganism concentration Ω(ζ) profile. Intensity of temperature is not good for the survival of microorganism concentration. Therefore, in the present case, the microorganism concentration Ω(ζ) increases with moderate temperature. Figure 38 projects that the the microorganism concentration Ω(ζ) decreases with amplifying the porosity parameter $\gamma_{3}$. In porous medium there is a resistance of holes to the flow of microorganisms so their progress is not significant in that case. Figure 39 illustrates that the non-dimensional microorganism concentration Ω(ζ) profile depreciates with the rising values of inertial parameter $\gamma_{4}$. This is due to the fact that the inertial parameter affects the motion of the microorganisms since the environment is not clear to them. Figure 40 reveals that microorganism concentration field Ω(ζ) amplifies with the chemical reaction parameter $\gamma_{5}$. Figure 41 shows that the microorganism concentration Ω(ζ) decreases against the Brownian motion parameter *Nb* because the propulsion of microorganisms is weak. Figure 42 demonstrates that by increasing thermophoresis parameter *Nt*, the microorganism concentration profile Ω(ζ) increases. This is due to the close relation of concentration with moderate temperature and it is expected that a higher thermophoresis parameter allows a deeper penetration of the microorganism concentration. This achieves enhancement in species in the nanofluid and elevates the motile microorganism concentration profile Ω(ζ). This indicates that a stronger thermophoresis effect induces a resistance in the mass diffusion. In Figure 43, the microorganism concentration field Ω(ζ) shows an upshot for the greater values of Lewis number *Le*. This happens because as the Lewis number *Le* increases, the viscous diffusion rate increases which in turn reduces the velocity at the surface and so elevates the density of the microorganisms. The effect of Schmidt number *Sc* on the microorganism concentration profile Ω(ζ) is projected in Figure 44. When the magnitude of Schmidt number *Sc* increases, the microorganism concentration profile Ω(ζ) decreases. This is attributed to the low propulsion of microorganisms. Figure 45 shows the effect of bioconvection Peclet number *Pe* on the microorganism concentration profile Ω(ζ). It indicates that the microorganism concentration profile Ω(ζ) grows large for different values of bioconvection Peclet number *Pe*. This is due to the fact that microorganisms diffuse more and more with larger values of bioconvection Peclet number *Pe*. The bioconvection Peclet number *Pe* helps to increase the speed of the microorganisms in respect of the nanofluid and so the density of the microorganisms is reduced near the surface. The behavior is highly influenced with an increase in the Lewis number *Le* which again helps in the reduction of the mass diffusivity. Figure 46 shows that by increasing magnetic field parameter *M*, the nondimensional motile density function profile Ω(ζ) decreases because the magnetic field opposes the flow due to Lorentz force.

## 7. Entropy Generation Analysis

**Figure 47:**
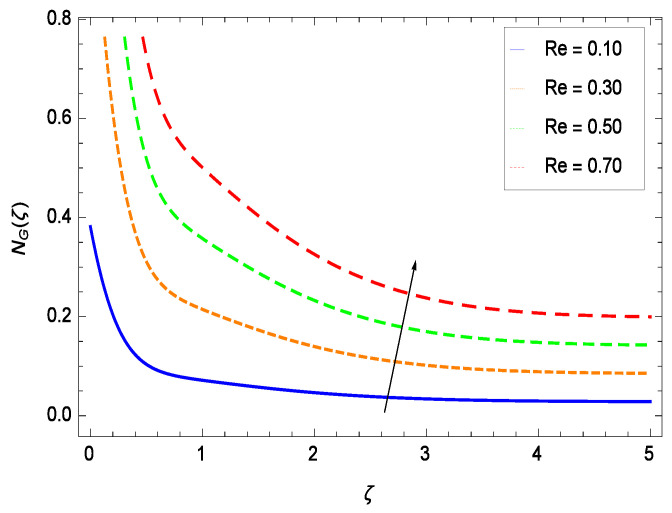
Effect on entropy generation rate for *h* = −1.50, *Br* = 0.40, *M* = 0.50, $\gamma_{6}$ = 0.60, $\theta_{w}$ = 0.70, $\phi_{w}$ = 0.80, $\Omega_{w}$ = 0.90 and different values of *Re*.

**Figure 48:**
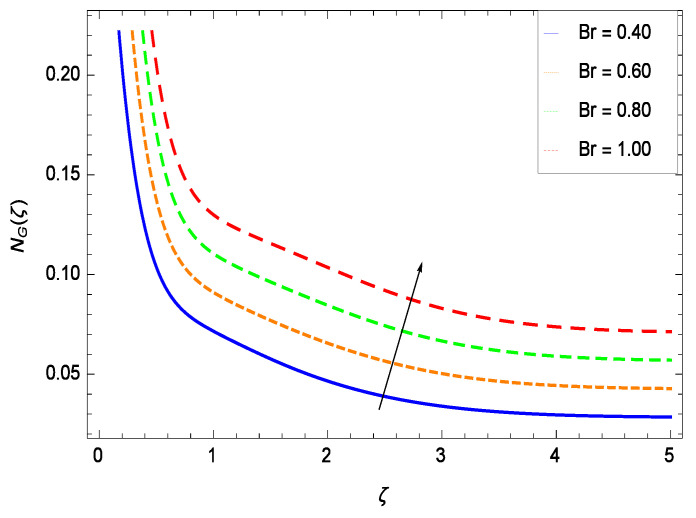
Effect on entropy generation rate for *h* = −1.50, *Re* = 0.10, *M* = 0.50, $\gamma_{6}$ = 0.60, $\theta_{w}$ = 0.70, $\phi_{w}$ = 0.80, $\Omega_{w}$ = 0.90 and different values of *Br*.

**Figure 49:**
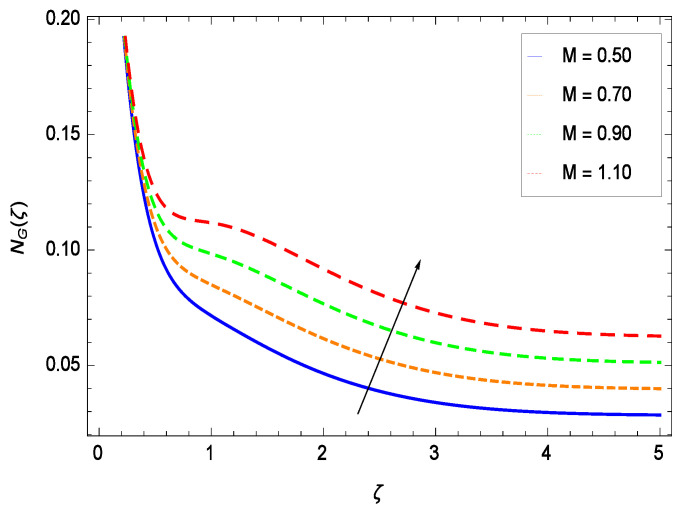
Effect on entropy generation rate for *h* = −1.50, *Re* = 0.10, *Br* = 0.40, $\gamma_{6}$ = 0.60, $\theta_{w}$ = 0.70, $\phi_{w}$ = 0.80, $\Omega_{w}$ = 0.90 and different values of *M*.

**Figure 50:**
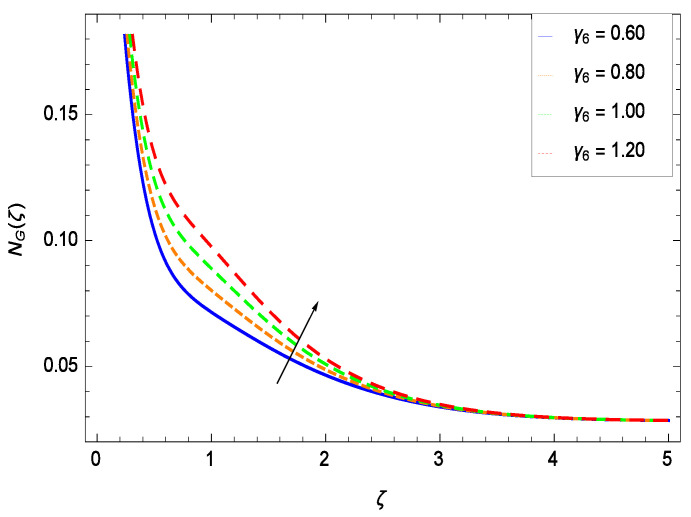
Effect on entropy generation rate for *h* = −1.50, *Re* = 0.10, *Br* = 0.40, *M* = 0.50, $\theta_{w}$ = 0.70, $\phi_{w}$ = 0.80, $\Omega_{w}$ = 0.90 and different values of $\gamma_{6}$.

**Figure 51:**
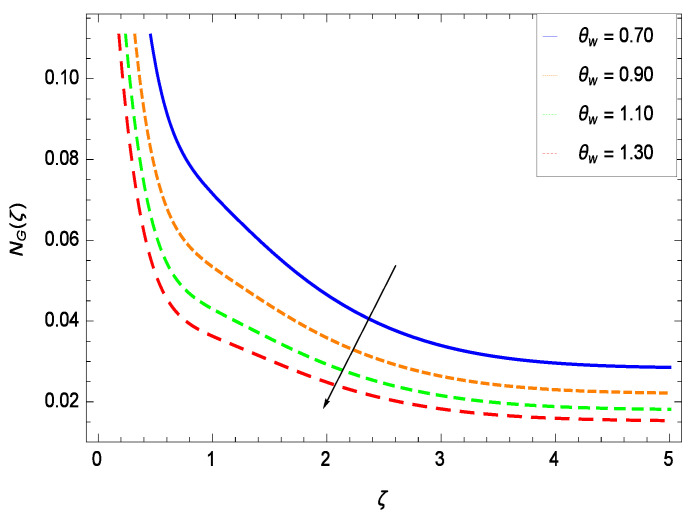
Effect on entropy generation rate for *h* = −1.50, *Re* = 0.10, *Br* = 0.40, *M* = 0.50, $\gamma_{6}$ = 0.60, $\phi_{w}$ = 0.80, $\Omega_{w}$ = 0.90 and different values of $\theta_{w}$.

**Figure 52:**
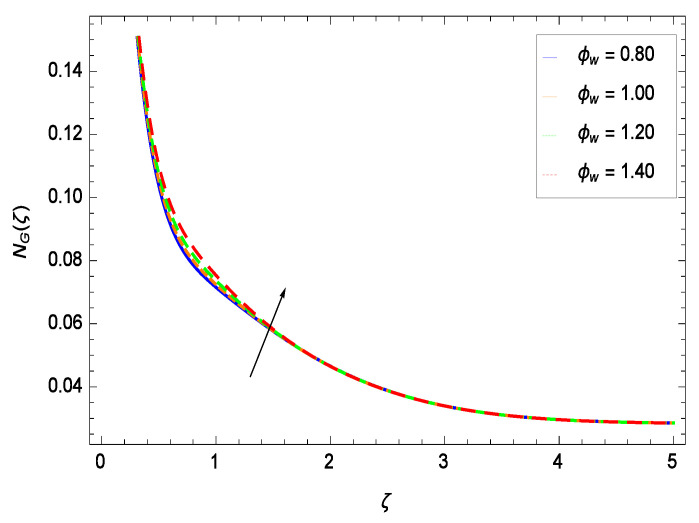
Effect on entropy generation rate for *h* = −1.50, *Re* = 0.10, *Br* = 0.40, *M* = 0.50, $\gamma_{6}$ = 0.60, $\theta_{w}$ = 0.70, $\Omega_{w}$ = 0.90 and different values of $\phi_{w}$.

**Figure 53:**
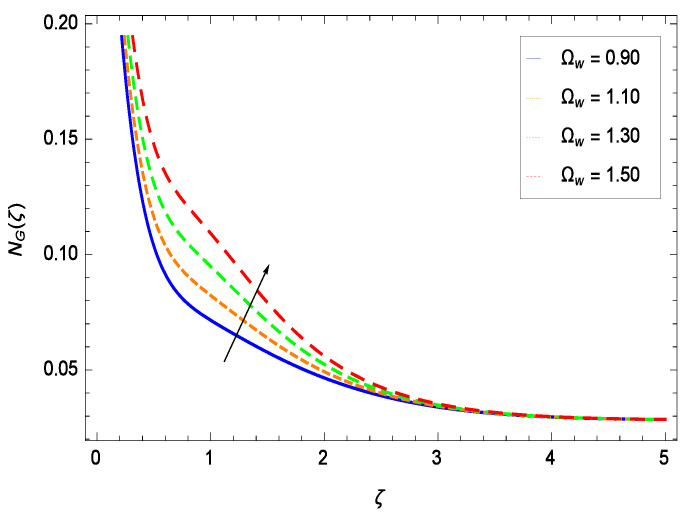
Effect on entropy generation rate for *h* = −1.50, *Re* = 0.10, *Br* = 0.40, *M* = 0.50, $\gamma_{6}$ = 0.60, $\theta_{w}$ = 0.70, $\phi_{w}$ = 0.80 and different values of $\Omega_{w}$.

It is necessary to study the irreversibility of the present system in terms of second-grade nanofluid, nanoparticles and microorganisms. Figure 47 shows that the irreversibility *N${}_{G}$*(ζ) of the system increases for high values of Reynolds number *Re*. Similarly, the values of *Br* make enhancement in entropy generation rate *N${}_{G}$*(ζ) which has the same increasing behavior in Figure 48. Different values of magnetic field parameter *M* in Figure 49 presents an increasing effect on the entropy generation rate *N${}_{G}$*(ζ). In Figure 50, the nondimensional diffusive constant parameter $\gamma_{6}$ assumes various high values (ranging from 0.50 to 1.20) with deep effects observations. $\gamma_{6}$ is related to the diffusivity. It shows that entropy generation rate *N${}_{G}$*(ζ) increases with increasing $\gamma_{6}$ where at the same time the temperature difference parameter $\theta_{w}$ reveals a unique activity, including the depreciation of entropy generation rate *N${}_{G}$*(ζ) in Figure 51. The formation of curves of the entropy generation rate *N${}_{G}$*(ζ) in Figure 52 is due to the various values of the concentration difference parameter $\phi_{w}$, a reactive species making increment in *N${}_{G}$*(ζ). In Figure 53, it is shown that microorganisms concentration difference parameter $\Omega_{w}$ favors the entropy generation rate.

## 8. Residual Errors

**Figure 54:**
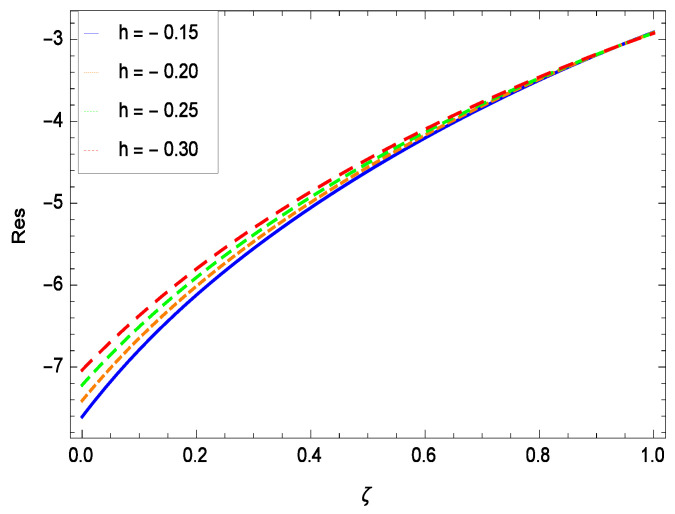
Residual errors graph of velocity profile for γ = 0.30, $\gamma_{1}$ = 0.30, $\gamma_{2}$ = 0.30, $\gamma_{3}$ = 0.30, $\gamma_{4}$ = 0.30, *Gr* = 0.40, *Nr* = 0.40, *Rb* = 0.50, *Nb* = 0.50, *Nt* = 0.60, *Le* = 0.60, *Sc* = 0.40, *Pe* = 0.40, *Pr* = 0.70, *M* = 0.60 and different values of *h*.

**Figure 55:**
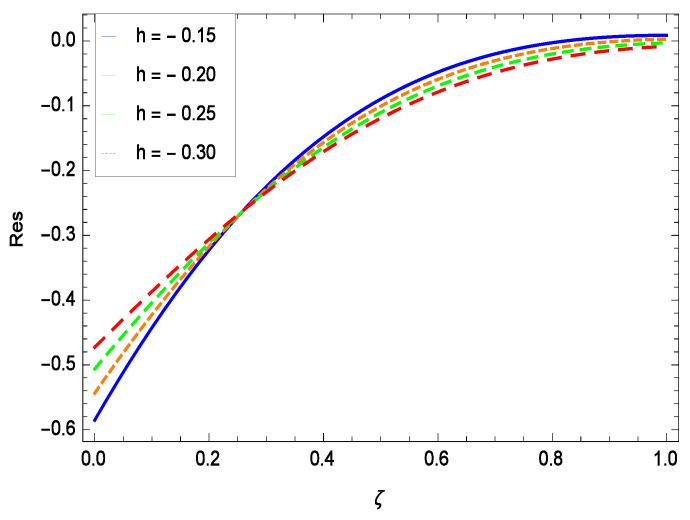
Residual errors graph of temperature profile for γ = 0.30, $\gamma_{1}$ = 0.30, $\gamma_{2}$ = 0.30, $\gamma_{3}$ = 0.30, $\gamma_{4}$ = 0.30, *Gr* = 0.40, *Nr* = 0.40, *Rb* = 0.50, *Nb* = 0.50, *Nt* = 0.60, *Le* = 0.60, *Sc* = 0.40, *Pe* = 0.40, *Pr* = 0.70, *M* = 0.60 and different values of *h*.

**Figure 56:**
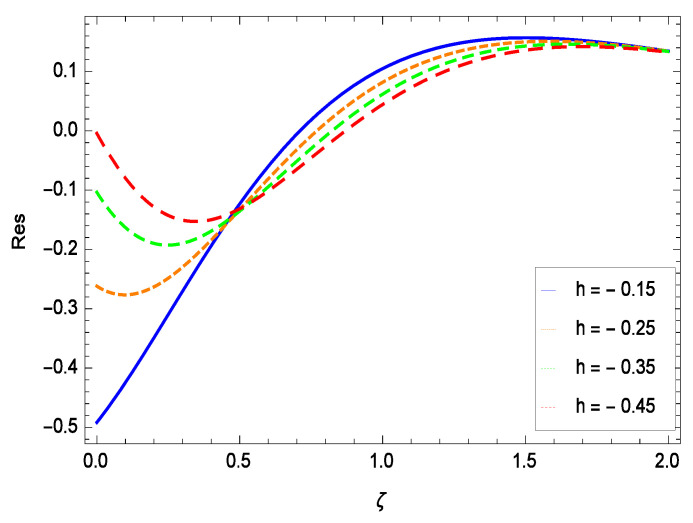
Residual errors graph of concentration profile for γ = 0.30, $\gamma_{1}$ = 0.30, $\gamma_{2}$ = 0.30, $\gamma_{3}$ = 0.30, $\gamma_{4}$ = 0.30, *Gr* = 0.40, *Nr* = 0.40, *Rb* = 0.50, *Nb* = 0.50, *Nt* = 0.60, *Le* = 0.60, *Sc* = 0.40, *Pe* = 0.40, *Pr* = 0.70, *M* = 0.60 and different values of *h*.

**Figure 57:**
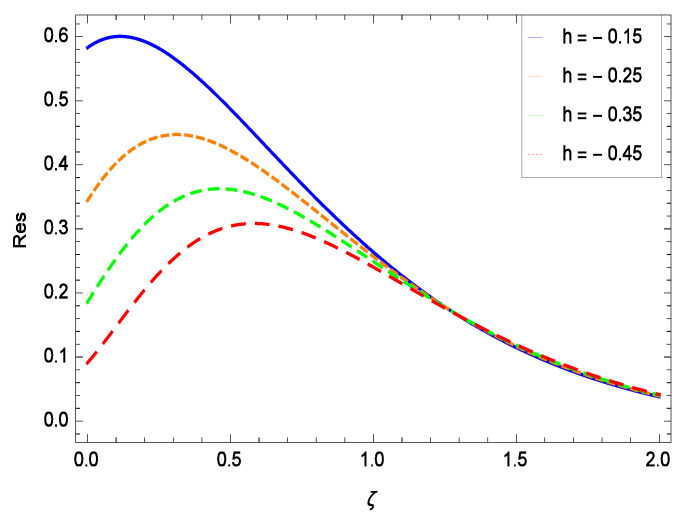
Residual errors graph of microorganism concentration profile for γ = 0.30, $\gamma_{1}$ = 0.30, $\gamma_{2}$ = 0.30, $\gamma_{3}$ = 0.30, $\gamma_{4}$ = 0.30, *Gr* = 0.40, *Nr* = 0.40, *Rb* = 0.50, *Nb* = 0.50, *Nt* = 0.60, *Le* = 0.60, *Sc* = 0.40, *Pe* = 0.40, *Pr* = 0.70, *M* = 0.60 and different values of *h*.

Residual errors are shown in the graphs to show the authentication of the problem. By fixing all the others parameters, different values are assigned to convergence control parameter *h* to show the effects in Figure 54, Figure 55, Figure 56 and Figure 57.

## 9. Conclusions

This article discusses the analytical solution of entropy generation and mixed convection in gravity-driven non-Newtonian second grade nanofluid flow containing both nanoparticles and gyrotactic microorganisms through a porous medium with a chemical reaction along a convectively heated vertical solid surface. The thin film considered in this work contains the mixture of nanoparticles and motile gyrotactic microorganisms [14]. The solution of the problem has been obtained by using analytical technique called Homotopy Analysis Method (HAM) for the velocity, temperature, concentration and microorganism concentration fields, which has been displayed in the diagrams. From Figure 6, Figure 7, Figure 8, Figure 9, Figure 10, Figure 11, Figure 12, Figure 13, Figure 14, Figure 15, Figure 16, Figure 17, Figure 18, Figure 19, Figure 20, Figure 21, Figure 22, Figure 23, Figure 24, Figure 25, Figure 26, Figure 27, Figure 28, Figure 29, Figure 30, Figure 31, Figure 32, Figure 33, Figure 34, Figure 35, Figure 36, Figure 37, Figure 38, Figure 39, Figure 40, Figure 41, Figure 42, Figure 43, Figure 44, Figure 45, Figure 46, Figure 47, Figure 48, Figure 49, Figure 50, Figure 51, Figure 52 and Figure 53, each figure displays the profound effect of the emerging parameters on the flow, heat transfer, nanoparticles concentration, microorganism concentration and entropy generation. Residual errors sketches are drawn in Figure 54, Figure 55, Figure 56 and Figure 57 to show the authentication of the HAM solution. The main findings of the study are summarized as following.

(i) The velocity *f*(ζ) depreciates for the porosity parameter $\gamma_{3}$, inertial parameter $\gamma_{4}$, bioconvection Rayleigh number *Rb* and magnetic field parameter *M* while it elevates for the second grade nanofluid parameter $\gamma_{1}$, reduced heat transfer parameter $\gamma_{2}$, chemical reaction parameter $\gamma_{5}$, buoyancy parameter *Gr*, buoyancy ratio parameter *Nr*, Lewis number *Le*, Schmidt number *Sc* and Prandtl number *Pr*.
(ii) The temperature θ(ζ) diminishes for the reduced heat transfer parameter $\gamma_{2}$, porosity parameter $\gamma_{3}$, inertial parameter $\gamma_{4}$, magnetic field parameter *M* and Prandtl number *Pr* while it elevates for the second grade nanofluid parameter $\gamma_{1}$, chemical reaction parameter $\gamma_{5}$ and thermophoresis parameter *Nt*.
(iii) The nanoparticles concentration ϕ(ζ) diminishes for second grade nanofluid parameter $\gamma_{1}$, reduced heat transfer parameter $\gamma_{2}$, thermophoresis parameter *Nt* and Prandtl number *Pr* while it elevates for the porosity parameter $\gamma_{3}$, inertial parameter $\gamma_{4}$, chemical reaction parameter $\gamma_{5}$, Brownian motion parameter *Nb*, Lewis number *Le* and magnetic field parameter *M*.
(iv) The microorganism concentration Ω(ζ) diminishes for the porosity parameter $\gamma_{3}$, inertial parameter $\gamma_{4}$, Brownian motion parameter *Nb*, Schmidt number *Sc* and magnetic field parameter *M* while it elevates for the second grade nanofluid parameter $\gamma_{1}$, reduced heat parameter $\gamma_{2}$, chemical reaction parameter $\gamma_{5}$, Lewis number *Le*, thermophoresis parameter *Nt* and bioconvection Peclet number *Pe*.
(v) Entropy generation rate *N${}_{G}$*(ζ) diminishes with temperature difference parameter $\theta_{w}$ while it elevates for Reynolds number *Re*, Brinkman number *Br*, magnetic field parameter *M*, diffusive constant parameter $\gamma_{6}$, nanoparticles concentration difference parameter $\phi_{w}$ and microorganism concentration difference parameter $\Omega_{w}$.
(vi) Residual errors graphs are self explanatory for the efficiency of HAM solution.

## Figures and Tables

**Figure 2:**
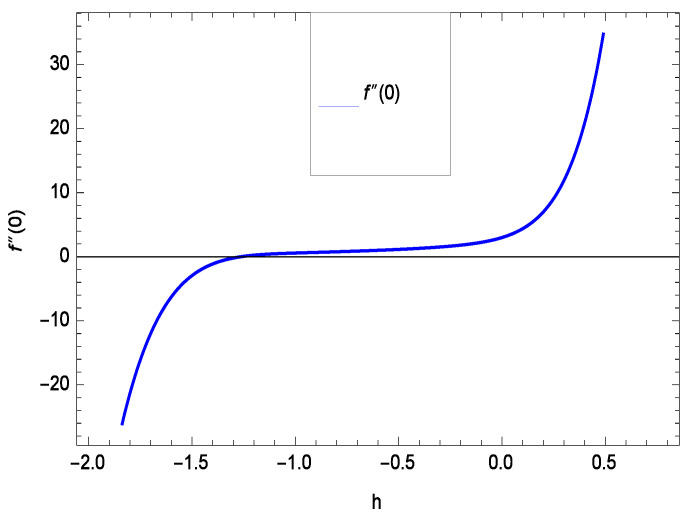
The *h* curve of *f*(ζ).

**Figure 3:**
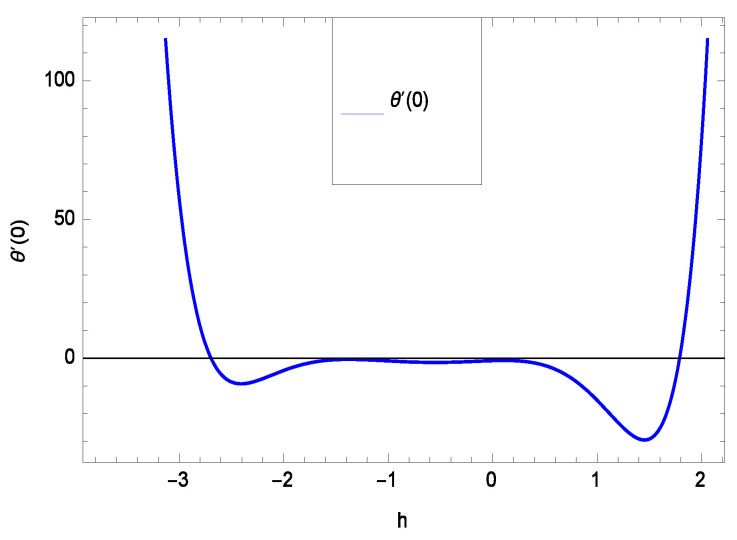
The *h* curve of θ(ζ).

**Figure 4:**
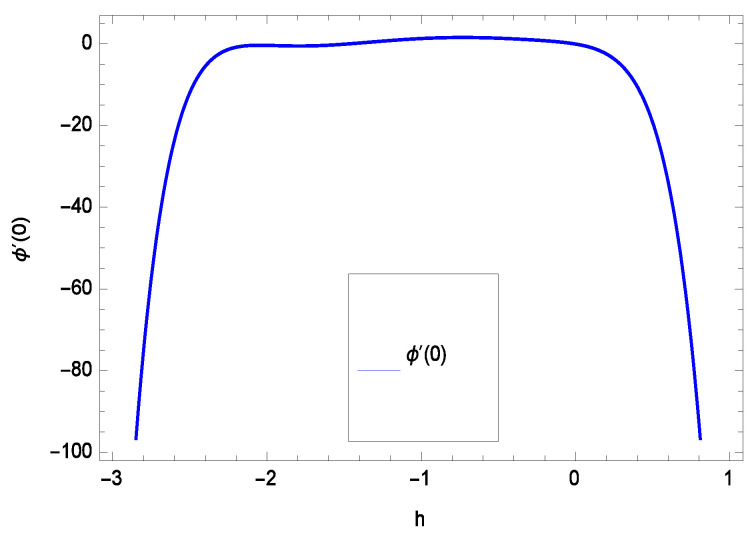
The *h* curve of ϕ(ζ).

**Figure 5:**
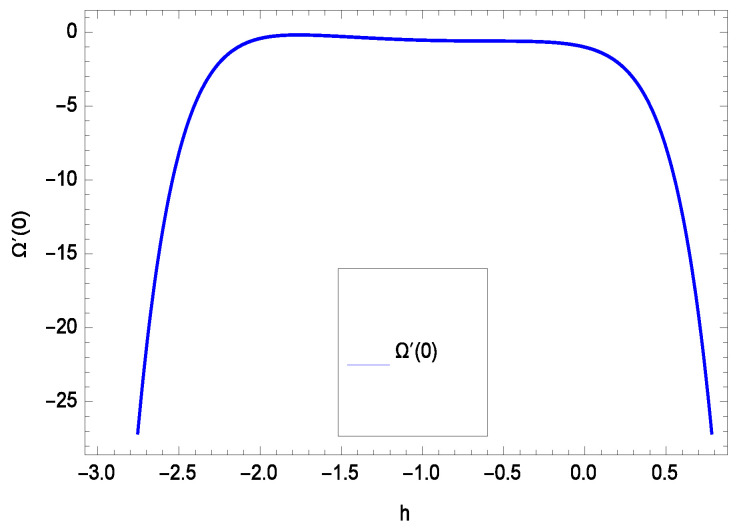
The *h* curve of Ω(ζ).

**Table 1:** Some recent developments related to the topic under investigation.

Author Names	Author Works	Some Outcomes
Khan et al. [1]	Entropy generation	Entropy generation increases with Reynolds number
Ishaq et al. [2]	Entropy generation	Entropy generation decreases with Eyring-Powell parameter
Hayat et al. [3]	Entropy generation	Entropy generation increases with Reynolds number
Khan et al. [4]	Bioconvection	Bioconvection decreases with reduced heat transfer parameter
Zuhra et al. [5]	Bioconvection	Gyrotactic microorganisms depreciates with magnetic field parameter
Khan [6]	Bioconvection	Stratification increases with second-grade fluid parameter
Palwasha et al. [7]	Bioconvection	Stratification increases with gravitational forces
Raees et al. [8]	Bioconvection	Bioconvection depends on upper plate
Zuhra et al. [9]	Bioconvection	Microorganisms decrease with increasing Brownian motion parameter
Pedley [10]	Instability	Instability of the system is due to microorganisms
Xu et al. [11]	Mixed convection	Nanoparticles favor the mixed convection
Aziz et al. [12]	Free convection	Instability of the system increases due to microorganisms
Bhatti et al. [13]	Chemical reaction	Mass transfer increases with chemical reaction
Raees et al. [14]	Bioconvection‘	Passively controlled nanofluid model provides better results
Ramzan et al. [15]	Chemical reaction	Mass transfer increases with chemical reaction
Anjalidevi [16]	Chemical reaction	Mass transfer increases with chemical reaction

**Table 2:** Various parameters in Equations (10)–(14).

Parameter Names	Symbols/Notations	Defined Values
Dimensionless second-grade nanofluid parameter	$\gamma_{1}$	$\frac{\alpha_{1}U}{4\mu_{f}x}$
Thermal Grashof number	*Gr*	$\frac{2\left(1-C_{\infty }\right)\rho_{f_{\infty }}g\beta \left(T_{f}-T_{\infty }\right)}{3a\rho_{f}}$
Buoyancy ratio parameter	*Nr*	$\frac{2\left(\rho_{p}-\rho_{f}\right)C_{\infty }g}{3a\rho_{f}}$
Bioconvection Rayleigh number	*Rb*	$\frac{2\left(N_{w}-N_{\infty }\right)g\gamma_{av}\Delta \rho }{3a\rho_{f}}$
Prandtl number	*Pr*	$\frac{\nu_{f}}{\lambda }$
Thermophoresis parameter	*Nt*	$\frac{\tau D_{T}\left(T_{f}-T_{\infty }\right)}{\lambda T_{\infty }}$
Brownian motion parameter	*Nb*	$\frac{\tau D_{B}C_{\infty }}{\lambda }$
Lewis number	*Le*	$\frac{\nu_{f}}{D_{B}}$
Schmidt number	*Sc*	$\frac{\nu_{f}}{D_{n}}$
Bioconvection Peclet number	*Pe*	$\frac{bW_{c}}{D_{n}}$
Reduced heat transfer parameter	$\gamma_{2}$	$\frac{h_{f}}{k_{1}}\sqrt{\frac{4x\nu_{f}}{3U}}$
Porosity parameter	$\gamma_{3}$	$\frac{4\nu_{f}}{3k_{0}U}$
Inertial parameter	$\gamma_{4}$	$\frac{4k_{0}^{F}}{3\sqrt{k_{0}}}$
Chemical reaction parameter	$\gamma_{5}$	$\frac{4k_{r}\nu \sqrt{x}}{3bD_{B}}$
Magnetic field parameter	*M*	$\frac{4\sigma B_{0}^{2}}{3\rho_{f}U}\sqrt{\frac{x}{2a}}$

## Abbreviations

The following abbreviations are used in this manuscript:

*x*	*x*-axis coordinate (m)
*y*	*y*-axis coordinate (m)
*u*	Velocity component along *x*-axis (m s${}^{-1}$)
*v*	Velocity component along *y*-axis (m s${}^{-1}$)
$\overset{s}{v}$	Average swimming velocity (m s${}^{-1}$)
*U*	Free stream velocity (m s${}^{-1}$)
*W${}_{c}$*	Maximum cell swimming speed (m s${}^{-1}$)
*a*	Constant
*b*	Chemotaxis constant
*B${}_{0}$*	Magnetic flux density (Tesla)
*Gr*	Thermal Grashof number
*M*	Magnetic field parameter
*Nr*	Buoyancy ratio parameter
*Rb*	Bioconvection Rayleigh number
*Nt*	Thermophoresis parameter
*Nb*	Brownian motion parameter
*Le*	Lewis number
*Sc*	Schmidt number
*Pe*	Bioconvection Peclet number
*Pr*	Prandtl number
*g*	Gravitational acceleration (m s${}^{-2}$)
*P*	Pressure (kg m${}^{-1}$ s${}^{-2}$)
*c${}_{p}$*	Specific heat at constant pressure (J kg${}^{-1}$ K${}^{-1}$)
*k*	Thermal conductivity (W/m K)
*T*	Temperature (K)
*T${}_{f}$*	Convective surface temperature (K)
*T${}_{\infty }$*	Ambient fluid temperature (K)
*h${}_{f}$*	Heat transfer coefficient
*C*	Nanoparticles concentration
*C${}_{\infty }$*	Ambient fluid concentration
*N*	Number density of motile microorganisms
*N${}_{w}$*	Wall concentration of microorganisms
*N${}_{\infty }$*	Ambient concentration of microorganisms
*N${}_{G}$*	Entropy generation number
*D*	Diffusivity
*D${}_{B}$*	Brownian diffusion coefficient
*D${}_{T}$*	Thermophoretic diffusion coefficient
*D${}_{n}$*	Diffusivity of microorganisms
*f*(ζ)	Dimensionless velocity
*L*	Characteristic length (m)
*R*	Ideal gas constant
*Re*	Reynolds number
*Br*	Brinkman number
$\alpha_{1}$	Normal stress moduli
σ	Electrical conductivity ((Ω·m)${}^{-1}$)
μ	Coefficient of viscosity (kg m${}^{-1}$ s${}^{-1}$)
ρ	Density (kg m${}^{-3}$)
ν	Kinematic viscosity (m${}^{2}$ s${}^{-1}$)
ψ	Physical stream function (m${}^{2}$ s${}^{-1}$)
β	Coefficient of volumetric volume expansion
Δ	Difference operator
λ	Thermal diffusivity of nanofluid (m${}^{2}$ s${}^{-1}$)
τ	Ratio of the heat capacitances of nanoparticle and base fluid
ζ	A scaled boundary layer coordinate
θ(ζ)	Dimensionless temperature
$\theta_{w}$	Dimensionless temperature difference
ϕ(ζ)	Dimensionless concentration
$\phi_{w}$	Dimensionless concentration difference
Ω(ζ)	Dimensionless microorganisms concentration
$\Omega_{w}$	Dimensionless microorganisms concentration difference
$\gamma_{av}$	Average volume of microorganisms (m${}^{-3}$)
$\gamma_{1}$	Dimensionless second grade nanofluid parameter
$\gamma_{2}$	Reduced heat transfer parameter
$\gamma_{3}$	Porosity parameter
$\gamma_{4}$	Inertial parameter
$\gamma_{5}$	Chemical reaction parameter
$\gamma_{6}$	Diffusive constant parameter due to nanoparticles concentration
$\gamma_{7}$	Non-dimensional positive number
$\gamma_{8}$	Diffusive constant parameter due to microorganisms concentration
av	Average
B	Brownian
c	Cell
p	Solid particles
r	Reaction
n	Properties related to microorganisms
f	Base fluid
o	Origin
x	Local value
w	Properties at the wall
*∞*	Fluid properties at ambient condition
Superscripts	
*s*	Swimming
′	Differentiation w. r. t. ζ
